# The Author’s Contributions to Echocardiography Literature (Part I—1978–1990) †

**DOI:** 10.3390/children7040032

**Published:** 2020-04-10

**Authors:** P. Syamasundar Rao

**Affiliations:** University of Texas-Houston McGovern Medical School, Children’s Memorial Hermann Hospital, 6410 Fannin Street, UTPB Suite # 425, Houston, TX 77030, USA; P.Syamasundar.Rao@uth.tmc.edu; Tel.: +1-713-500-5738; Fax: +1-713-500-5751

**Keywords:** Doppler echocardiography, ventricular septal defect, contrast echocardiography, left ventricular hypoplasia syndromes, left ventricular function, ventricular muscle mass, afterload reduction, balloon valvuloplasty, balloon angioplasty, atrial septal defect, aortic coarctation

## Abstract

The author has undertaken multiple echocardiographic studies during his academic career; most of these were published in peer-reviewed journals. These studies include an evaluation of the role of echocardiography in the estimation of left-to-right shunt in isolated ventricular septal defects, an examination of the utility of contrast echocardiography in the diagnosis of anomalous connection of the right superior vena cava to the left atrium, a description of pitfalls in M-mode echocardiographic assessment of the aortic root in left ventricular hypoplasia syndromes, reviews of echocardiographic evaluation of left ventricular function, study of the role of contrast echocardiography in the evaluation of hypoxemia following open heart surgery, a quantification of left ventricular muscle mass by m-mode echocardiography in children, an examination of race and sex related differences in echocardiographic measurements in children, study of cardiac size and function in patients with sickle cell disease, an examination of afterload reduction in the management of primary myocardial disease, study of the utility of echo-Doppler studies in the evaluation of the results of balloon pulmonary valvuloplasty, study of the usefulness of Doppler in the prediction of pressure gradients in valvar pulmonary stenosis, a review of Doppler echocardiography in noninvasive diagnoses of heart disease, echo-Doppler studies of the evaluation of the results of balloon angioplasty of aortic coarctation, study of the value of Doppler in the prediction of pressure gradients across coarctation of the aorta, and a characterization of foramen ovale and transatrial Doppler velocity patterns in the normal fetus.

## 1. Introduction

When the author was named as Interim Director of Pediatric Echocardiography Laboratory at Children’s Memorial Hermann Hospital/University of Texas-Houston Medical School, Houston, Texas in 2006, some of the staff critiqued this appointment, as the author was considered to be an interventionist pediatric cardiologist. The author responded to this critique by sending them a list of the his echo publications [1,2,3,4,5,6,7,8,9,10,11,12,13,14,15,16,17,18,19,20,21,22,23,24,25,26,27,28,29,30,31,32,33]. It may be worthwhile reviewing the author’s professional echocardiography experience.

At the time of the author’s fellowship training in 1969/1970 at Stanford University Medical Center, Palo Alto, California, there were only two echocardiography recording machines in the United States: one was at University of Indiana, Indianapolis, Indiana and the other at Stanford University. The author was exposed to echocardiography to evaluate mitral valve motion and pericardial effusion on M-mode tracings. During subsequent fellowship training at Case-Western Reserve University and the University of California at Los Angeles, the echo machines had not yet arrived at the respective institutions. By 1972, when the author joined the Faculty at the Medical College of Georgia, Augusta, Georgia, there were indeed echocardiography facilities at that institution. Shortly thereafter, Dr. Wesley Covitz (who was trained with Richard Meyer, one of the premier pediatric echocardiographers of that time) joined our faculty at Georgia and provided the author with additional understanding of the principles and practice of M-mode echocardiography, thereby adding to the author’s knowledge base. This resulted in the author’s active participation in echocardiography research at that time [1,2,3,4,5]. Two-dimensional (2D) echocardiography was coming in to vogue as the author was leaving Medical College of Georgia to take a position at the King Faisal Specialist Hospital & Research Center in Riyadh, Saudi Arabia. There were portable echocardiography machines (Apagee) at that institution which the author could use in a clinic setting and perform 2D studies of children following their formal evaluations by history, physical examination, electrocardiogram (ECG) and chest x-ray (norms at that time). This provided the author with experience and expertise to teach sonographers and other trainees. As the author gained expertise in 2D, suddenly, Doppler echocardiography surfaced. The author had to learn this modality as well. Then, as the author was leaving Riyadh to join the Faculty at University of Wisconsin, Madison, Wisconsin, the color Doppler technique was introduced, which again, the author had to learn. In this review, the author’s contributions to the echocardiography literature will be discussed.

## 2. Estimation of Left-to-Right Shunt in Isolated Ventricular Septal Defects by Echocardiography

**Figure 1:**
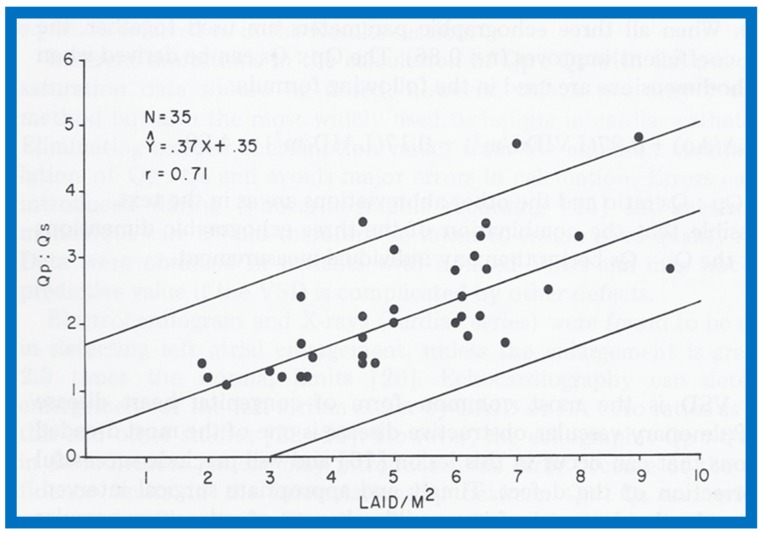
Scattergram demonstrating the relationship of left atrial internal dimension/m^2^ (LAID/m^2^) with pulmonary-to-systemic flow ratio (Qp:Qs) in patients with isolated ventricular septal defects. The central line is regression line and the parallel lines demarcate the confidence intervals. The number of patients (N), regression equation and correlation coefficient (r) are shown in the insert at the top left. Reproduced from Rees AH, Rao P.S., et al. [1].

**Figure 2:**
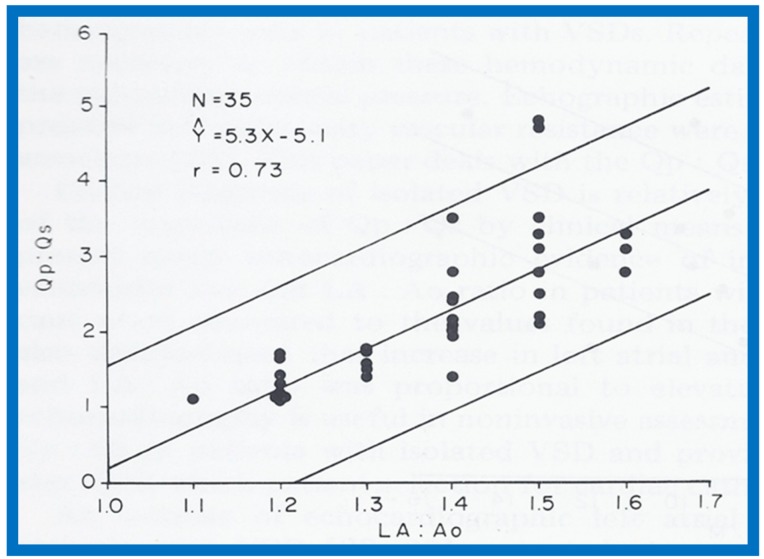
Scattergram demonstrating the relationship of the left atrium to aortic root ratio (LA:Ao) with pulmonary-to-systemic flow ratio (Qp:Qs) in patients with isolated ventricular septal defects. The central lines and insert are as in Figure 1. Reproduced from Rees AH, Rao P.S., et al. [1].

**Figure 3:**
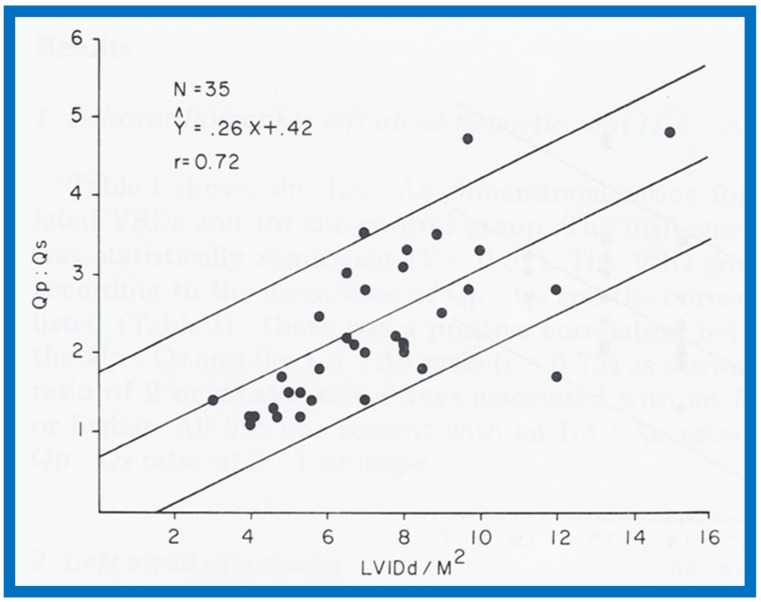
Scattergram demonstrating the relationship of left ventricular internal dimension/m^2^ (LVIDd/m^2^) with pulmonary-to-systemic flow ratio (Qp:Qs) in patients with isolated ventricular septal defects. The central lines and insert are as in Figure 1. Reproduced from Rees AH, Rao P.S., et al. [1].

In the mid-1970s, in an attempt to noninvasively quantify the degree of left-to-right shunt in infants and children with isolated ventricular septal defects (VSDs), we compared the cardiac catheterization data with echocardiographic values (echo performed within 24 h of cardiac catheterization) in 35 babies; 35 children with normal hearts by physical examination and ECG were used as controls [1]. The size of the left atrium (LA), the LA to aortic root (Ao) ratio and the size of the left ventricle (LV) in the VSD group were larger (*p* < 0.01) than those of control subjects (for actual values, see Table I of [1]). In the VSD group, the echo values were increased in proportion to the catheterization-measured pulmonary to systemic flow ratio (Qp:Qs), with r values ranging from 0.71 to 0.73 (Figure 1, Figure 2 and Figure 3). In addition, Qp:Qs greater than 2:1 was always present with a LA:Ao ratio larger than 1.4:1. It was found that the combination of these echo parameters resulted in better predictions of Qp:Qs than any of the echo parameters used alone. 

We concluded that the echocardiographic technique is useful in estimating Qp:Qs and in the clinical assessment of patients with isolated VSD [1]. The author is pleased that these observations remain valid, even more than 40 years after the publication of our study, and the addition of 2D and Doppler studies have increased the value of echocardiography in the assessment of VSD patients so that cardiac catheterization and angiography is rarely required prior to surgery at the present time.

## 3. Contrast Echocardiography in the Diagnosis of Anomalous Connection of the Right Superior Vena Cava to the Left Atrium 

**Figure 4:**
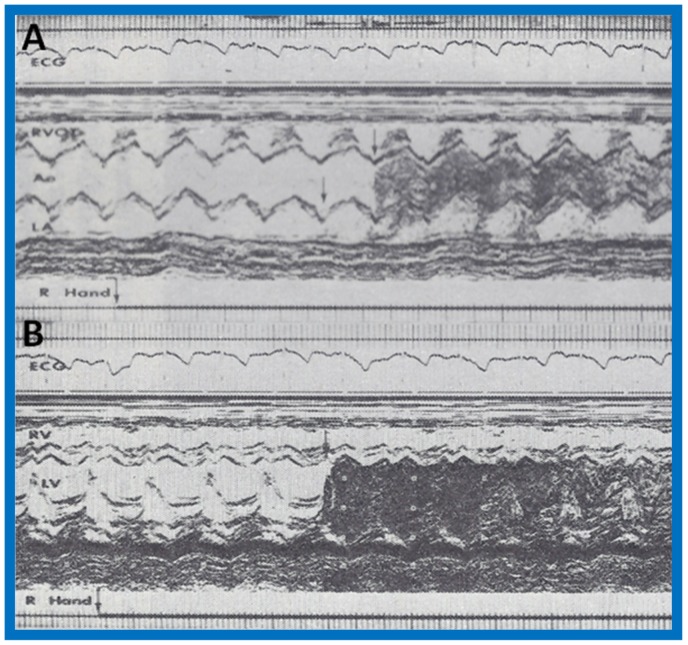
Selected M-mode recordings from the parasternal short axis view of the left atrium (LA), aorta (Ao), and right ventricular outflow tract (RVOT) while injecting agitated saline into veins of the right (R) hand (**A**) demonstrating the appearance of contrast echoes in the LA (arrow) first and then Ao (arrow). Similar tracings of the left ventricle (LV) and right ventricle (RV) (**B**) demonstrate appearance of contrast echoes in the LV (arrow) without contrast in the RV. Similar findings were seen while injecting agitated saline into veins of the left hand. These recordings indicate drainage of the superior vena cava into the left atrium. The start of agitated saline injection is marked with arrows at the bottom of each tracing. ECG, electrocardiogram. Reproduced (modified) from Truman T.A., Rao P.S., Kulungara R.J. [2].

**Figure 5:**
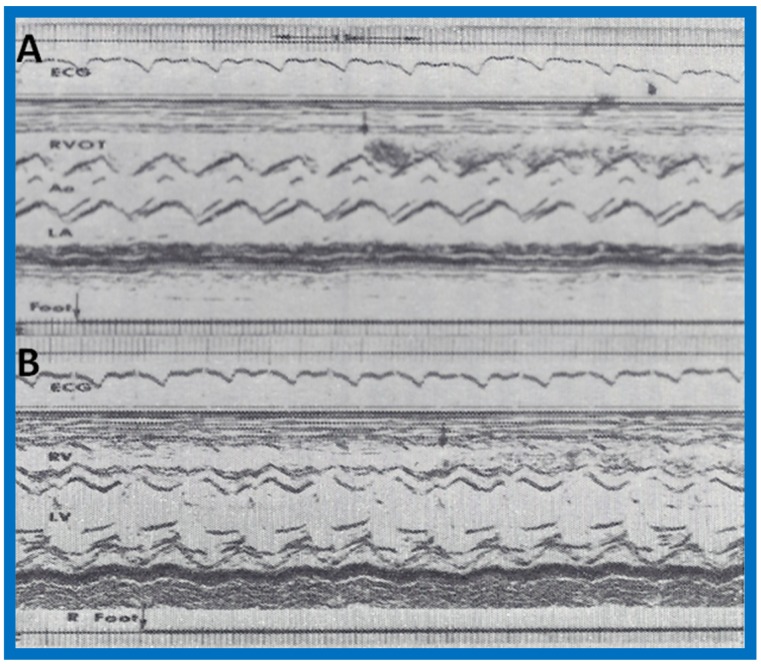
Recordings similar to Figure 4 were made while injecting agitated saline into the veins of the feet and these reveal contrast in the RVOT (**A**) and RV (**B**) (arrows in **A** and **B**), suggesting that the inferior vena cava drains normally into the right atrium (not shown) and RV. The start of agitated saline injection is marked with arrows at the bottom of each tracing. ECG, electrocardiogram. Reproduced (modified) from Truman T.A., Rao P.S., Kulungara R.J. [2].

An infant without abnormal cardiac findings other than cyanosis was studied by contrast echocardiography (by injection of agitated saline) and an anomalous systemic venous connection to the left atrium was suspected [2]. A moderate degree of cyanosis was noted by the pediatrician at 4 months of age and the baby was referred to the author for cardiac evaluation. Arterial blood gas analysis revealed a PO_2_ of 31 torr in room air, confirming the clinical finding of cyanosis. The PO_2_ did not increase following the administration of 100% oxygen for 15 min. Physical examination did not reveal any other abnormalities. The electrocardiogram (ECG) was normal, as was her M-mode echocardiogram. Agitated saline injection into a vein on the dorsum of each hand resulted in opacification of the left atrium, left ventricle and aorta (Figure 4A,B) without visualization of the right heart structures, suggesting anomalous drainage of the superior vena cava into the left atrium. Injection into a vein in the right foot resulted in opacification of the right heart without visualization of the left heart (Figure 5A,B), suggesting normal drainage of the inferior vena cava into the right atrium. 

**Figure 6:**
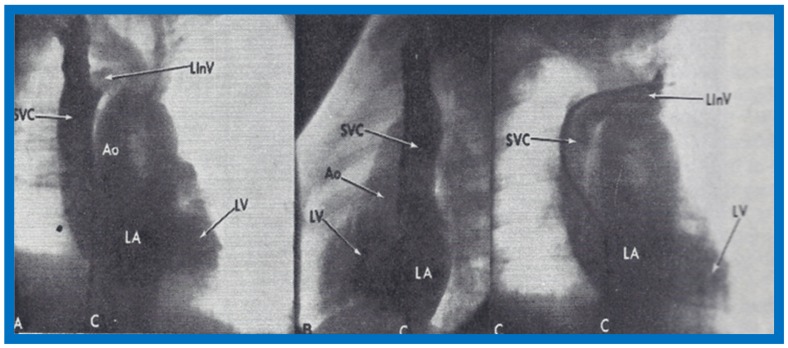
Angiographic frames from injections into the right innominate (**A**,**B**) and left innominate (LInV) (**C**) veins demonstrating direct opacification of the right superior vena cava (SVC), left atrium (LA), left ventricle (LV) and aorta (Ao) without opacification of the right heart structures. C, Catheter. Reproduced from Truman T.A., Rao P.S., Kulungara R.J. [2].

At the age of 8 months, cardiac catheterization with selective cineangiography was performed. The left atrium (LA) was catheterized via a tight patent foramen ovale, and the catheter was advanced from the LA into the superior vena cava (SVC) and into both right and left innominate veins. The right heart saturations were low (60–62%) without any evidence for a step up. The pulmonary venous saturations were normal (98%) with a step down in the left atrium and left ventricle (79%). The pressures in all cardiac chambers were normal (See Table 1 of [2]). Selective cineangiography into the right (Figure 6A,B) and left (Figure 6C) innominate veins confirmed the diagnosis of anomalous connection of the right SVC to the LA. No persistent left SVC was identified. Because of a lack of other symptoms and only mild polycythemia (hemoglobin of 15 gm%), surgical correction was deferred at that time with a plan for surgery at a later date. 

An extensive review of the literature revealed only four published cases of anomalous connection of the right SVC to the LA as of the time of our review [2]. Ages at diagnosis were 2 to 34 years, with our subject being 8 months-old. All of them presented with cyanosis and diagnosis was made by angiography, except for our case, which was initially diagnosed by contrast echocardiography and later confirmed by angiography. The differential diagnosis of cyanosis (and arterial desaturation) without any other abnormal cardiac findings includes pulmonary arteriovenous fistula, connection of pulmonary artery to the LA and of course anomalous systemic venous connection to the left atrium. In pulmonary arteriovenous fistula and connection of pulmonary artery to the LA, the contrast would first appear in the right ventricle before the LA, LV and Ao. In addition, differential contrast patterns at the site of injection into the veins of the upper and lower extremities should not be present with pulmonary artriovenous fistula and connection of pulmonary artery to the LA. The associated discussion also included a review of the possible embryological origin of anomalous systemic venous connection to the left atrium [2].

**Figure 7:**
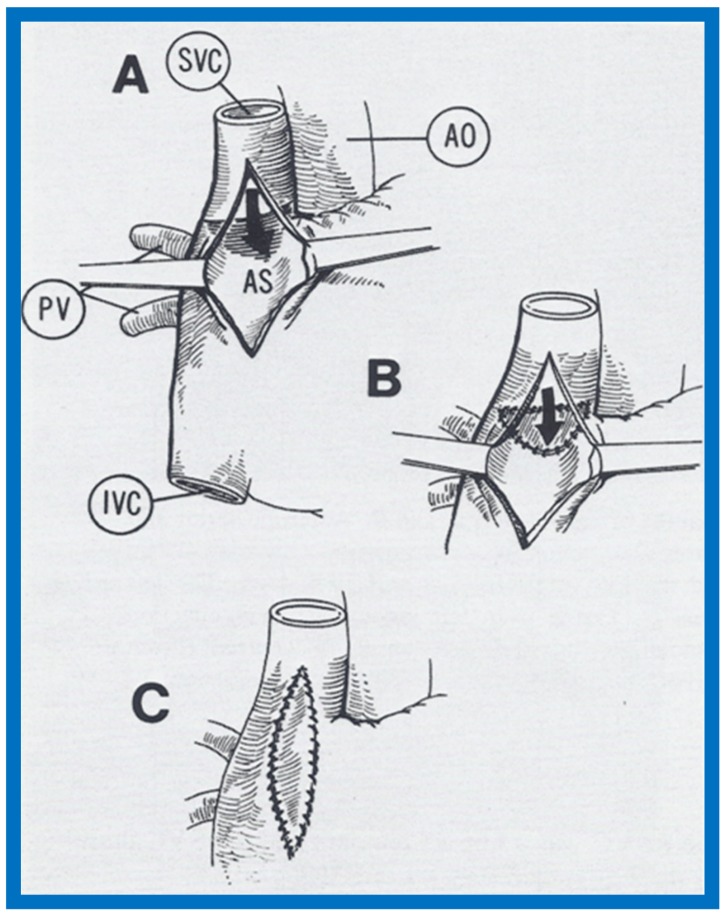
Artist’s portrayal of surgical correction of the superior vena cava (SVC) into the left atrium. (**A**) After incising the SVC, blood is seen going behind the atrial septum (AS) into the left atrium (solid arrow in **A**). The shadowed area was resected. (**B**) A pericardial patch was sewn in such a way as to divert the SVC flow into the right atrium (solid arrow in **B**). (**C**) A pericardia patch was placed to enlarge the SVC-right atrial junction. Ao, aorta; IVC, inferior vena cava; PV, pulmonary veins. Reproduced from Alpert B.S., Rao P.S., et al. [3].

**Figure 8:**
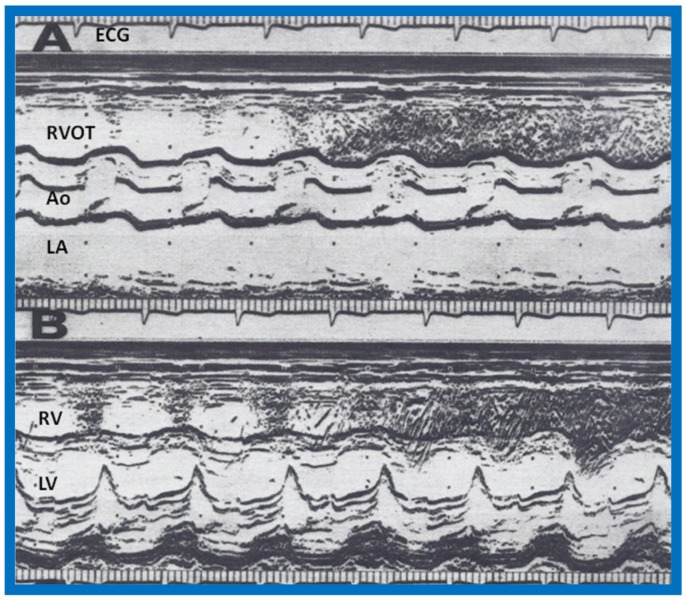
Following surgical correction, recordings similar to Figure 4 were made while injecting agitated saline into the veins of the hand; these revealed contrast in the right ventricular outflow tract (RVOT) (**A**) and right ventricle (RV) (**B**), suggesting that the superior vena cava now drains normally into the right atrium (not shown) and RV. Ao, aorta; ECG, electrocardiogram; LA, left atrium; LV, left ventricle. Modified from Alpert B.S., Rao P.S., et al. [3].

At the age of twenty-six months, surgical repair was performed under cardiopulmonary bypass, as described in Figure 7 [3]. Repeat contrast study following surgical correction showed opacification of right heart structures without contrast detected in the left heart after injection of agitated saline into the arm veins (Figure 8), indicating the positive outcome of the surgery [3]. The discussion focused on methods of surgical repair performed in the other three cases reported prior to our report and the usefulness of contrast echocardiography in diagnosing several types of congenital heart defects [3]. 

**Figure 9:**
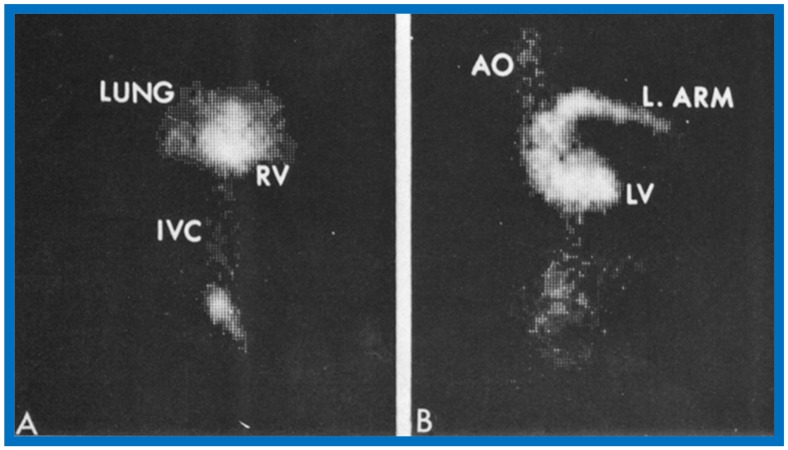
First pass radionuclide angiographic study of the patient with anomalous connection of the right superior vena cava to the left atrium. (**A**) When radionuclide material (tecnetium-99m macro-aggregated albumin) was injected into a vein in the foot, the inferior vena cava (IVC), right ventricle (RV) and lungs were seen, indicating normal drainage of the IVC into the right atrium. (**B**) When the injection was made into a vein of the left hand, the left (L) arm vein, the superior vena cava (not marked), left atrium (not marked), left ventricle (LV) and aorta (AO) were seen, indicating anomalous entry of the superior vena cava into the LA. Reproduced from Rao P.S. [34].

In an editorial communication published later [34], apart from a discussion of the lack of gender predilection for the prevalence of this rare anomaly, the differential diagnosis of cyanosis without any other abnormal cardiac findings with the use of noninvasive contrast echo studies, surgery to treat this anomaly and postoperative evaluation, we presented another noninvasive method to diagnose this condition, namely first pass radionuclide angiographic study (Figure 9).

## 4. Pitfalls in Echocardiographic Assessment of the Aortic Root in Left Ventricular Hypoplasia Syndromes

**Figure 10:**
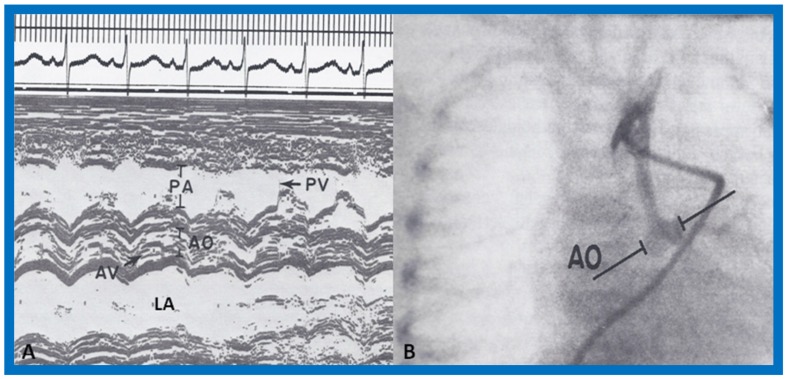
(**A**) Selected M-mode recordings from the parasternal short axis view of the left atrium (LA), aorta (Ao), and pulmonary artery (PA), demonstrating near normal sized aorta in a baby with hypoplastic left heart syndrome. (**B**) Selected cine frame from aortic arch angiogram in postero-anterior projection of the same infant shown in A, demonstrating hypoplastic ascending aorta and seemingly normal aortic sinuses, explaining the finding of near normal sized aorta in A. AV, aortic valve; PV, pulmonary valve. Modified from Covitz W, Rao P.S., et al. [4].

A comparison of M-mode echocardiograms and angiograms in six infants with left ventricular hypoplasia indicated that (a) a normal-sized aortic root echogram (Figure 10) may be seen in babies with mitral atresia, (b) an identifiable mitral valve echogram may be present in mitral atresia, and (c) it may be difficult to make accurate measurements of the echocardiographic left ventricular dimensions, with a tendency for overestimation [4]. It was concluded that the echocardiographer should be aware of the described pitfalls during M-mode echo diagnosis of left ventricular hypoplasia [4]. It should be noted that this study was conducted prior to introduction of 2D echocardiography.

## 5. Reviews: Echocardiographic Evaluation of Left Ventricular Function

We reviewed echocardiographic evaluations of the left ventricular function in two papers in early 1980s [5,6]. We began with a review of determinants of ventricular function, namely, preload or ventricular end-diastolic volume, contractile state of the myocardium, which is the innate force of the myocardial contraction and is independent of the load on the ventricle and afterload, or intraventricular systolic tension during ventricular ejection. Then, it was proposed that ventricular functional abnormalities in children could be grouped into four major pathophysiologic categories: 1. Volume overloading of the ventricle, as seen in ventricular septal defect, patent ductus arteriosus and mitral and aortic insufficiency; 2. Pressure overloading, as seen in aortic stenosis and systemic hypertension; 3. Ventricular under loading, as seen in mitral stenosis and pericardial tamponade; and 4. Primary disturbance of the contractile state of the myocardium, as seen in cardiomyopathy. 

**Figure 11:**
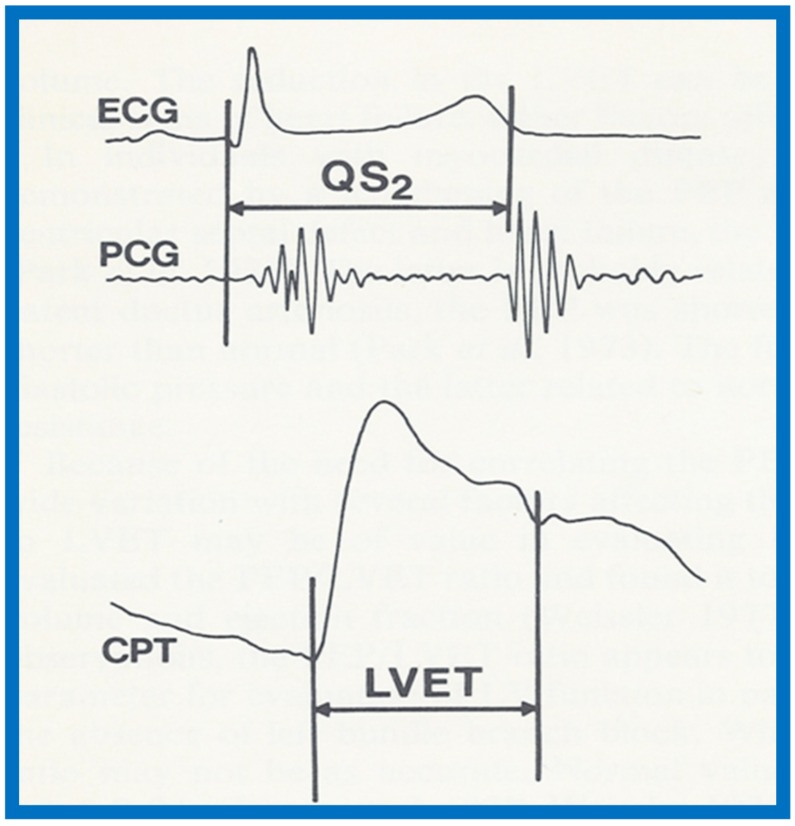
Simultaneous recording of the electrocardiogram (ECG), phonocardiogram (PCG) and carotid pulse trace (CPT) to illustrate measurement of left ventricular ejection time (LVET) and Q wave of the ECG to S_2_ (QS_2_). The preejection period (PEP) is calculated by subtracting LVET from QS2. Reproduced from Rao P.S. [6].

**Figure 12:**
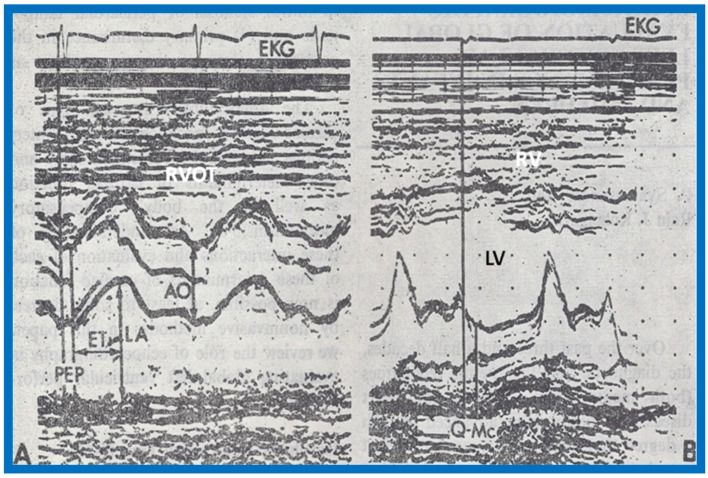
**(A**) Selected M-mode recordings from the parasternal short axis view of the left atrium (LA), aorta (Ao), and right ventricular outflow tract (RVOT) illustrating measurements of preejection period (PEP) and ejection time (ET). (**B**) Selected M-mode recordings from the parasternal short axis view of the left ventricle (LV) and right ventricle (RV) showing mitral valve closure and Q-Mc (Q wave of the electrocardiogram (EKG) to mitral valve closure). Isovolumic contraction time (ICT) is calculated by subtracting Q-Mc from PEP, ensuring that both recordings are made from cardiac cycles of identical length. Modified from Rao P.S., Kulangara R.J. [5].

**Figure 13:**
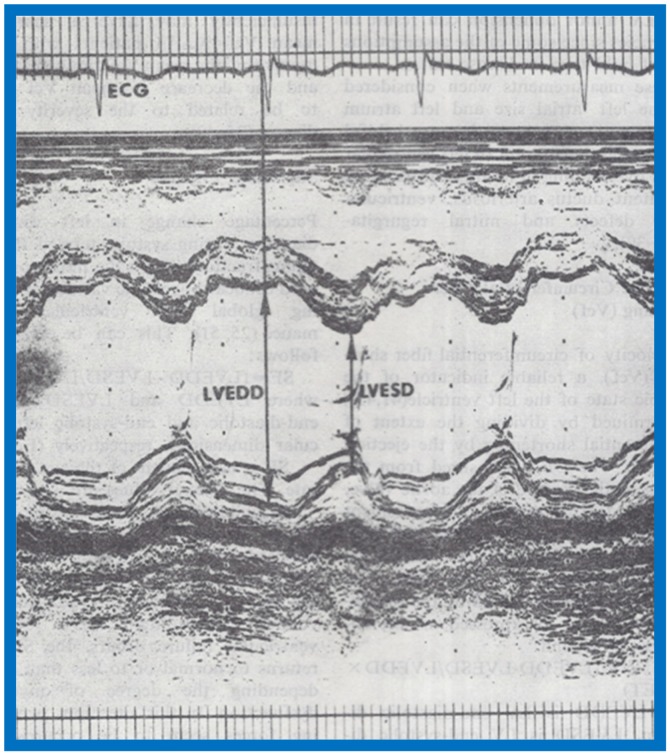
Selected M-mode recording from the parasternal short axis view of the left ventricle at the tips of the mitral valve illustrating measurements of left ventricular end-diastolic (LVEDD) and end-systolic (LVESD) dimensions to calculate shortening fraction (see Table 1). Reproduced from Rao P.S., Kulangara R.J. [5].

We presented the methods of measurements/calculations of systolic time intervals (Figure 11 and Figure 12), left ventricular volumes, LV shortening fraction (Figure 13), LV velocity of circumferential fiber shortening (Vcf), percent thickening of the LV posterior wall (Table 1) and their utility in the evaluation of LV function. This was followed by a discussion of diastolic function of the LV, other indices of LV function, LV function in aortic stenosis, LV function in LV volume overloading conditions and congestive cardiomyopathy. Then, newer methods (as of early 1980s) of assessing LV function by two-dimensional echocardiography, Doppler ultrasound-measured aortic blood flow velocity and radio-nuclide ejection fraction were briefed. The papers were concluded by outlining the value and limitations of noninvasive technology, as they were at that time [5,6].

## 6. Echocardiographic Features of Tricuspid Atresia

**Figure 14:**
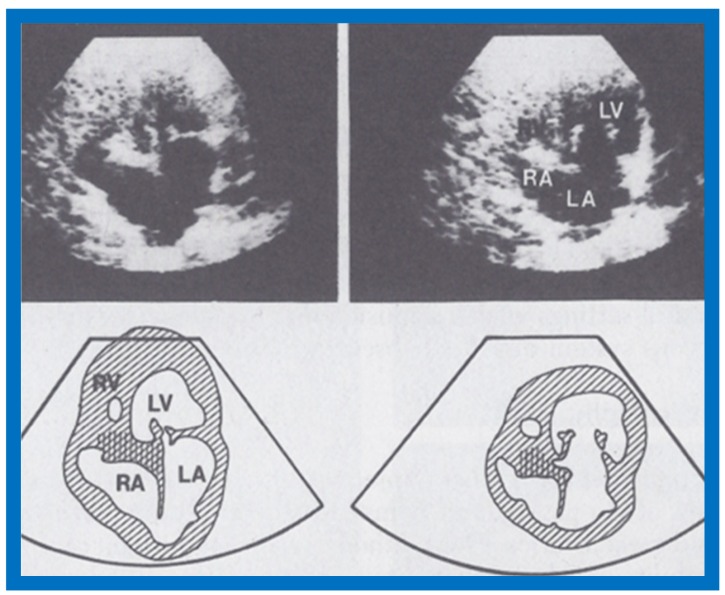
Selected video frames from apical four-chamber view of a two-dimensional (2-D) echocardiographic study demonstrating dense band of echoes between the right atrium (RA) and hypoplastic right ventricle (RV). Line drawings are shown beneath the 2D frames. Note that the mitral valve is closed in the left image while it is open in the right image. The atretic tricuspid valve echoes remain unchanged. LA, left atrium; LV, left ventricle. Reproduced from Covitz W., Rao P.S. [7].

The M-mode and two-dimensional echocardiographic features of tricuspid atresia were discussed in the author’s first book on tricuspid atresia, written in collaboration with his colleague, Dr Wesley Covitz at the Medical College of Georgia [7]. The two-dimensional (2D) echocardiographic pictures in 1982 were crude (Figure 14) [7]; however, they represented the state of the art echocardiography machines of that time. 

**Figure 15:**
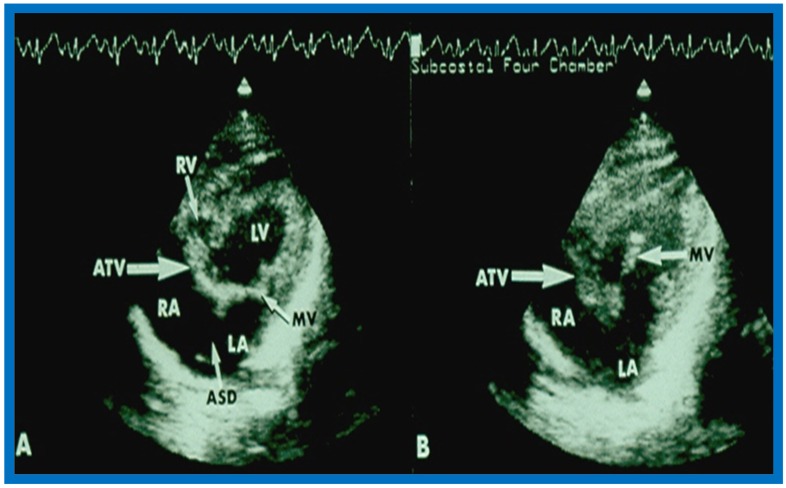
Selected video frames from subcostal four-chamber view of a two-dimensional (2-D) echocardiographic study demonstrating atretic tricuspid valve (ATV) (thick arrow), represented by a dense band of echoes between the right atrium (RA) and hypoplastic right ventricle (RV). In (**A**), the mitral valve (MV) is closed, while in (**B**), it is open. Note the improvement from the pictures shown in Figure 14. LA, left atrium; LV, left ventricle. Reproduced from Rao P.S. [35].

**Figure 16:**
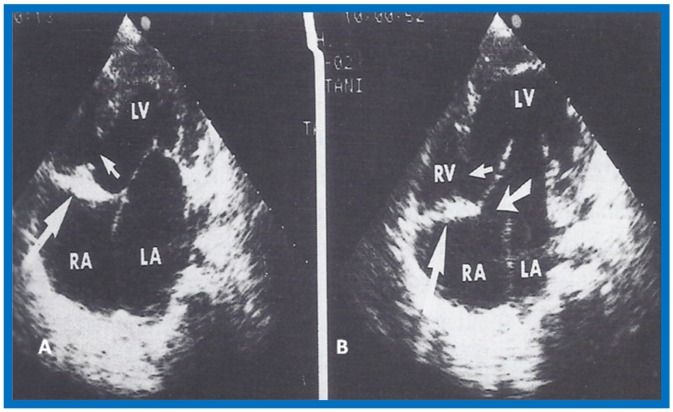
Apical four-chamber view pictures of another infant with tricuspid atresia (large arrows in **A**,**B**) with ostium primum atrial septal defect (slanted arrow in **B**). Note small right ventricle (RV) and a ventricular septal defect (small arrows in **A** and **B**). LA, left atrium; LV, left ventricle; RA, right atrium. Reproduced from Covitz W., Rao P.S. [36].

Shortly thereafter, better images (Figure 15 and Figure 16) became available [35,36].

**Figure 17:**
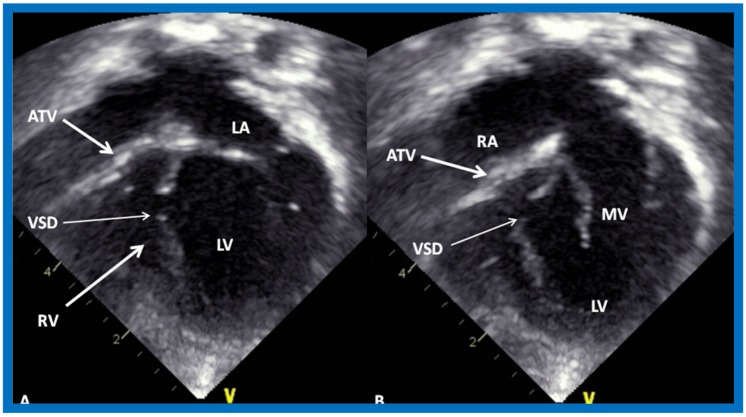
Selected video frames from apical four-chamber, 2-dimensional echocardiographic views of a neonate with tricuspid atresia showing an enlarged left ventricle (LV), a small right ventricle (RV) and a dense band of echoes at the site where the tricuspid valve echo should be (ATV) (thick arrow) with closed (**A**) and open (**B**) mitral valve. A moderate sized ventricular septal defect (VSD) (thin arrows) is shown. LA, Left atrium; RA, Right atrium. Reproduced from Rao P.S. [39].

**Figure 18:**
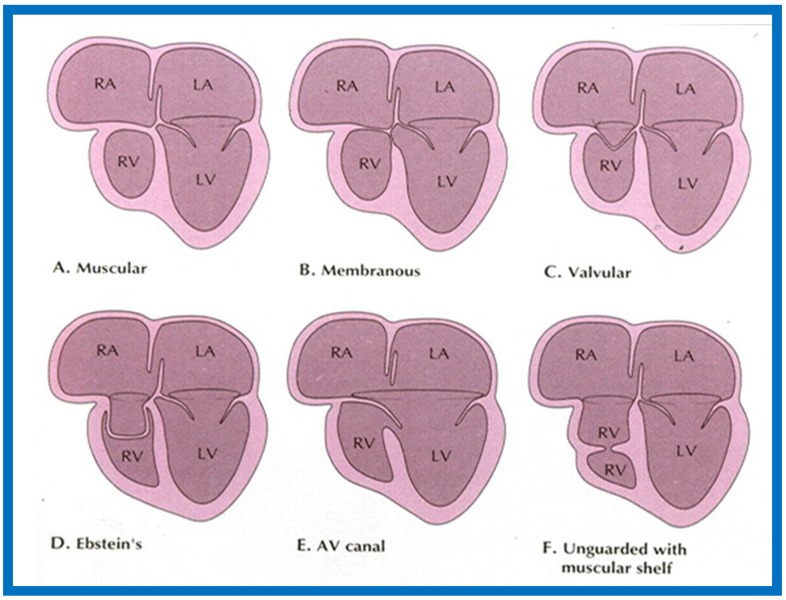
Classification based on morphology of the tricuspid valve is shown diagrammatically. (**A**) classic muscular type is shown with a thick band of tissue at the location where the tricuspid valve should be. (**B**) Membranous type, (**C**) Valvular type with fused valve leaflets, (**D**) Ebstein’s type with fused valve cusps along with Ebstein type of downward displacement of the tricuspid valve, (**E**) Atrioventricular (AV) canal (septal defect) type with common AV leaflet sealing off the right ventricle (RV) from the right atrium (RA), and (**F**) Unguarded muscular shelf type where the RV is divided into inlet and outlet portions by a muscular shelf. LA, left atrium; LV, left ventricle. Reproduced from Rao P.S. [40].

More recently, the author reviewed the echo-Doppler evaluation of tricuspid atresia [37,38,39]; these findings will be presented. M-mode echocardiography, while not diagnostic, is useful for evaluating the size of the left atrium (LA) and left ventricle (LV) and LV function. On 2D echocardiography, the atretic tricuspid valve is visualized directly as a dense band of echoes at the site where the tricuspid valve should be in the most frequent muscular type, as shown in Figure 14, Figure 15, Figure 16 and Figure 17. This anatomy is better demonstrated in apical and subcostal four-chamber views than in other views. The other types, namely membranous, valvular, Ebstein’s, atrioventricular septal and unguarded valve with muscular shelf (Figure 18) [40], are rare and may also be recognized on 2D echocardiography.

**Figure 19:**
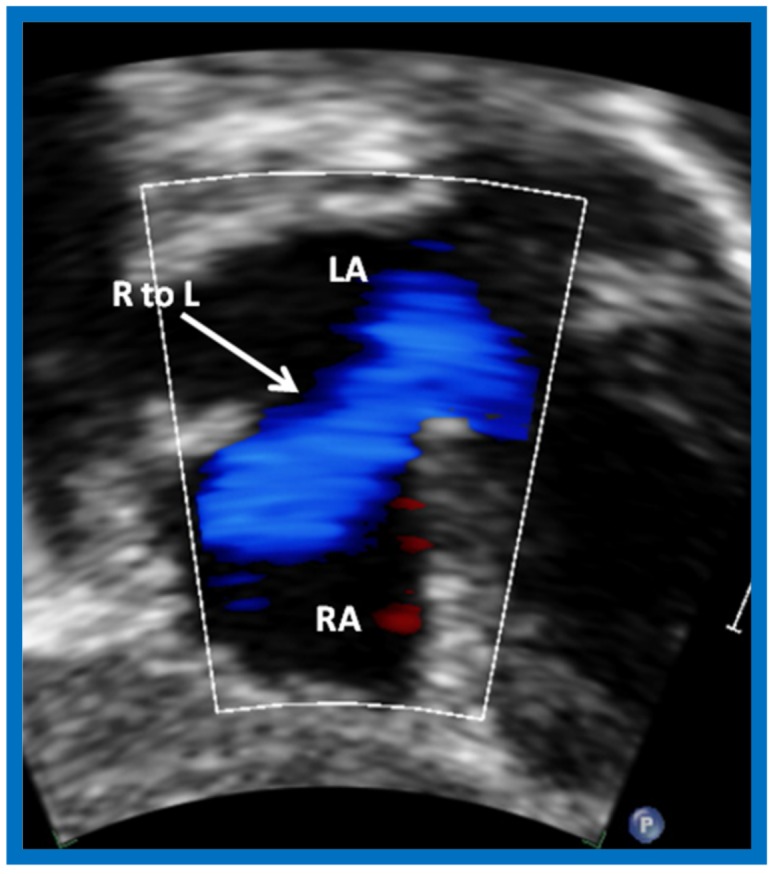
Selected video frame from subcostal view of a neonate with tricuspid atresia demonstrating right-to-left (R to L) shunt (arrow) across the interatrial communication. LA, Left atrium; RA, Right atrium. Reproduced from Rao P.S. [39].

Following the demonstration of the atretic tricuspid valve, the sizes of the cardiac chambers were evaluated both by M-mode (Z scores) and 2D echocardiography; enlarged RA, LA and LV, and a small RV, were seen (Figure 14, Figure 15, Figure 16 and Figure 17). Pulsed (not shown) and color Doppler (Figure 19) studies were helpful in illustrating the right-to-left shunt across the patent foramen ovale or atrial septal defect. Contrast study using agitated saline with 2D imaging clearly demonstrated successive opacification of the RA, LA, LV and then the RV in that order, but such a study is not necessary for diagnosis.

**Figure 20:**
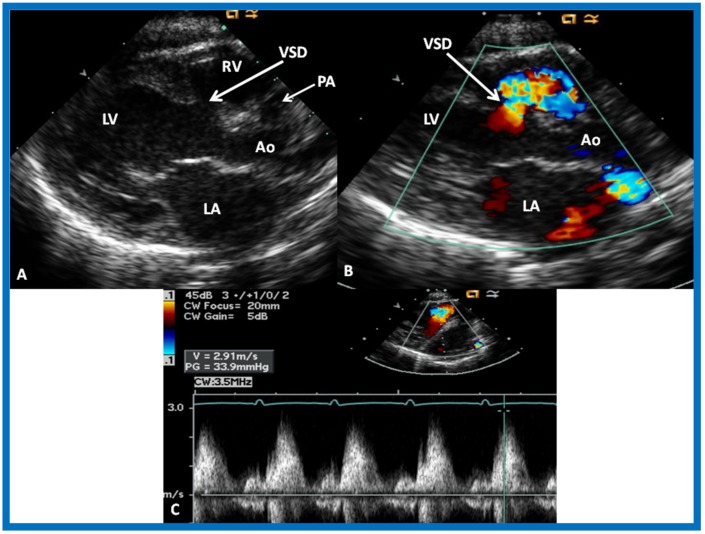
Selected video frames from precordial long axis views of a neonate with tricuspid atresia with normally related great arteries demonstrating enlarged left atrium (LA) and left ventricle (LV), a small right ventricle (RV) and a moderate sized ventricular septal defect (VSD) (thick arrow) on 2D (**A**) and color flow (**B**) imaging. Turbulent flow (**B**) with a Doppler flow velocity of 2.91 m/s by continuous wave Doppler (**C**) suggests some restriction of the VSD. Ao, Aorta; PA, pulmonary artery. Reproduced from Rao P.S. [39].

**Figure 21:**
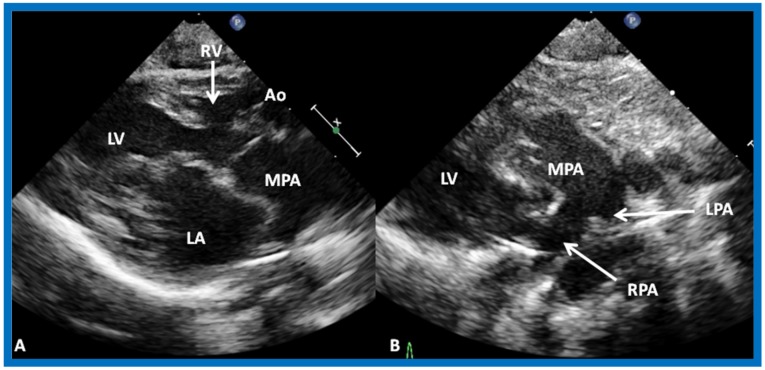
(**A**) Selected video frame from precordial long axis views of a neonate with tricuspid atresia and transposition of the great arteries demonstrating the left atrium (LA), left ventricle (LV), a very small right ventricle (RV) and a moderate sized ventricular septal defect (not marked). The vessel coming off of the LV is traced in (**B**) and shown to bifurcate into left (LPA) and right (RPA) pulmonary arteries, confirming that this is the main pulmonary artery (MPA). Ao, Aorta. Reproduced from Rao P.S. [39].

**Figure 22:**
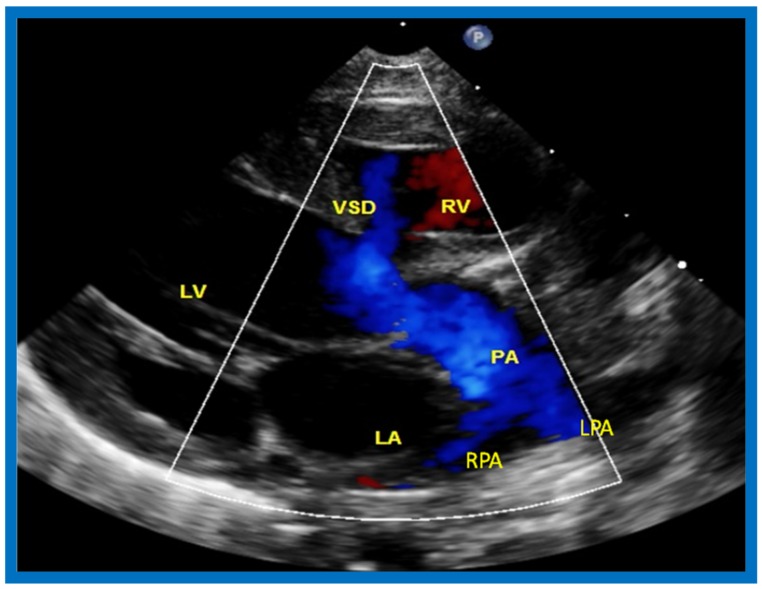
Selected video frame from precordial long axis view with color flow mapping of another neonate with tricuspid atresia and transposition of the great arteries illustrates the left atrium (LA), left ventricle (LV), a small right ventricle (RV) and a moderate sized ventricular septal defect (VSD). The vessel coming off of the LV bifurcates into left (LPA) and right (RPA) pulmonary arteries. Reproduced from Rao P.S. [39].

**Figure 23:**
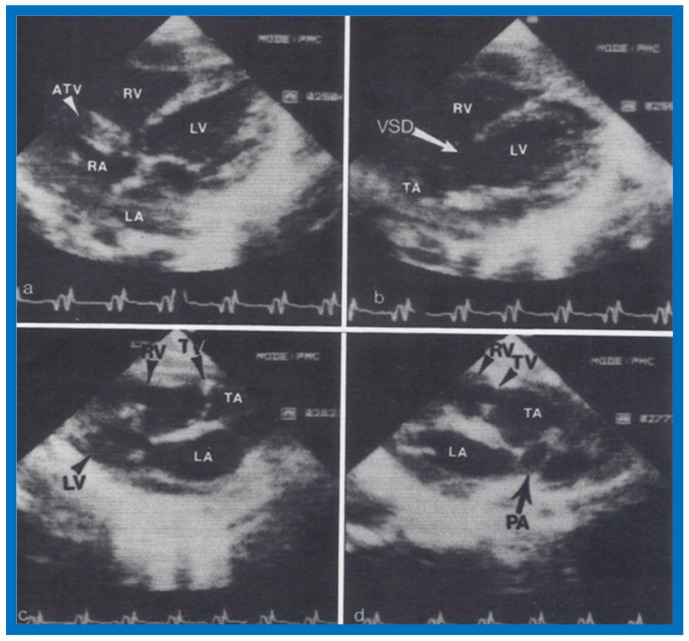
Two-dimensional echocardiographic video frames demonstrating (**a**) atretic tricuspid valve (ATV) between the right atrium (RA) and right ventricle (RV), (**b**) a large subtruncal ventricular septal defect (VSD), (**c**) thickened and somewhat domed truncal valve (TV) leaflets, and (**d**) origin of the pulmonary artery (PA) from the posterior aspect of the truncus arteriosus (TA). LA, Left atrium; LV, left ventricle. Reproduced from Rao P.S., et al. [22].

**Figure 24:**
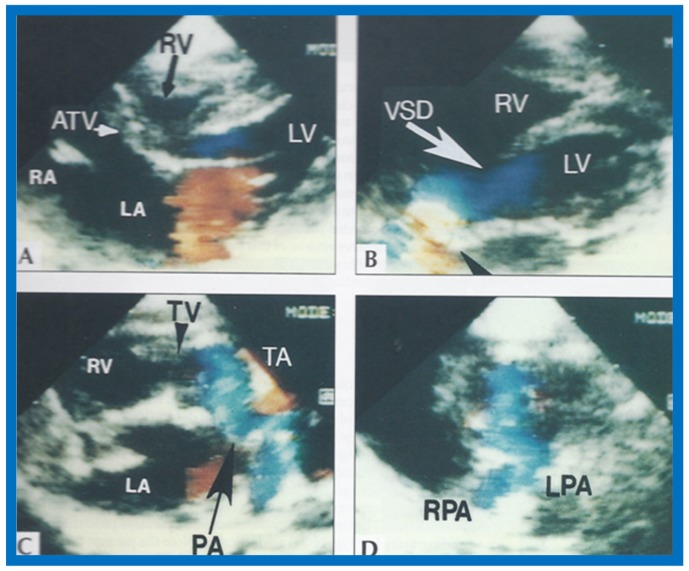
Video frame from a two-dimensional echocardiographic and color Doppler study demonstrating (**A**) atretic tricuspid valve (ATV) between the right atrium (RA) and right ventricle (RV) and blood flow from the left atrium (LA) into the left ventricle (LV) across the mitral valve. The RV (arrow) is very small and hypoplastic. (**B**) LV and RV with a large ventricular septal defect (VSD) below the truncus arteriosus (TA). Turbulent flow across the truncal valve suggests truncal valve stenosis. (**C**) origin of the pulmonary artery (PA) from the TA by color flow (arrow), and (**D**) division of right (RPA) and left (LPA) pulmonary arteries from the PA (labeled in d) in a short-axis view. TV, truncal valve leaflets. Reproduced from Rao P.S., et al. [22].

The relationship of the great arteries is examined next in order to classify them into various types [41]. The relationship of the great arteries is established by following the vessels arising from the ventricles until pulmonary bifurcation or aortic arch. In Type I patients with normally related great arteries, the aorta arises from the LV (Figure 20), while in Type II patients with transposition of the great arteries, the PA arises from the LV (Figure 21 and Figure 22). In Type III patients, it may be little more difficult to assign the great artery relationship, and sometimes angiography is needed. In type IV with truncus arteriosus, the limited data [22] suggest that this can be done by echocardiography (Figure 23 and Figure 24).

Further subdividing into subtypes a (pulmonary atresia), b (pulmonary stenosis or hypoplasia), and c (normal pulmonary arteries) is done by examining the pulmonary arteries irrespective of type. 

Then, the ventricular septum is evaluated; the ventricular septum is intact in most Type Ia cases. In children with Type I (normally related great arteries), the VSD supplies pulmonary blood flow (Figure 20) while in patients with Type II (transposition the great arteries), the VSD allows blood to flow into the systemic circuit (Figure 21 and Figure 22). In Type I patients, the VSD is demonstrated by 2D (Figure 20A) and the left to-right shunt across it by color (Figure 20B), pulsed and CW (Figure 20C) Doppler signals. Interrogation of the right ventricular outflow tract and pulmonary artery region is performed; peak Doppler flow velocity across the right ventricular outflow tract and pulmonary valve is helpful in identifying obstruction across these sites. The Doppler data from the VSD and RVOT are also helpful in estimating of pulmonary artery pressures. In these Type I babies, the 2D size of the VSD and the peak Doppler flow velocity across it are useful in quantifying the size of the VSD (Figure 20); the higher the VSD Doppler velocity, the smaller the defect. However, in patients with pulmonary hypertension, severe infundibular or valvar pulmonary stenosis, the VSD Doppler velocities do not reflect the size of the VSD. Barring these exceptions, right ventricular and pulmonary arterial pressure may be estimated using modified Bernoulli equation (RV/PA systolic pressure = systolic BP − 4V^2^). 

**Figure 25:**
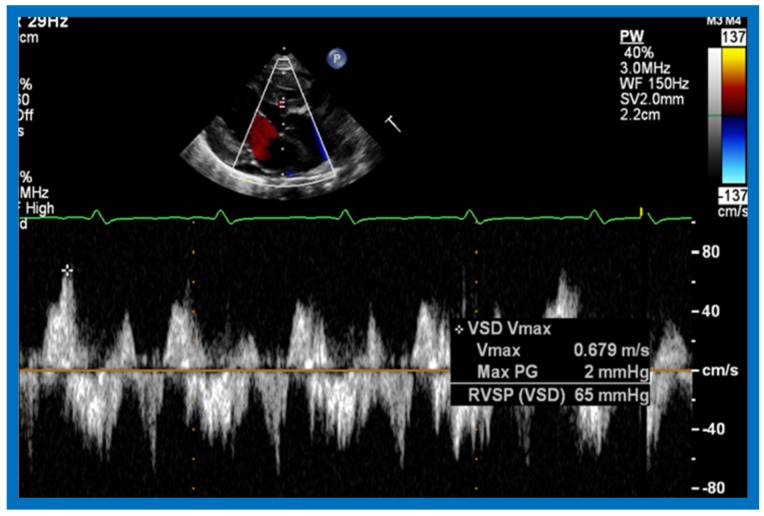
Selected video frame of continuous wave Doppler across the ventricular septal defect of the same baby shown in Figure 22. Low velocity flow across the ventricular septal defect suggests that the defect is nonobstructive. Reproduced from Rao P.S. [39].

**Figure 26:**
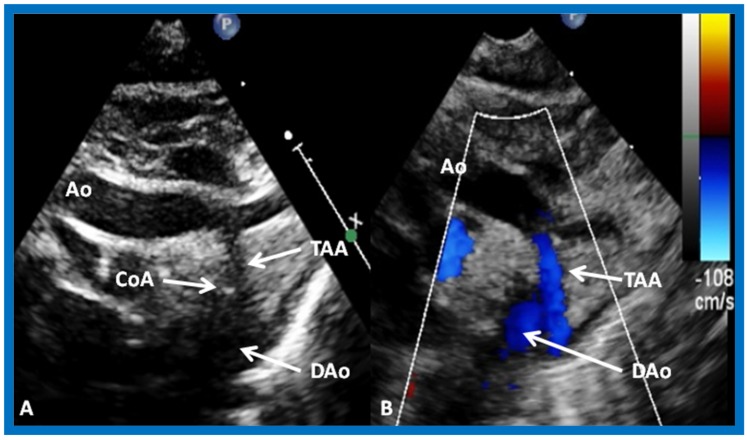
Selected video frames from suprasternal notch views of the aortic (Ao) arch in 2D (**A**) and color flow (**B**) images of a neonate with tricuspid atresia and transposition of the great arteries demonstrating coarctation of the aorta (CoA) and hypoplastic transverse aortic arch (TAA). The association of CoA with tricuspid atresia plus transposition of the great arteries is well known. DAo, descending aorta. Reproduced from Rao P.S. [39].

In Type II patients, the VSD may be small, causing obstruction to blood flow to the systemic circuit; therefore, the size of the VSD should be ascertained by 2D (Figure 21 and Figure 22), color Doppler (Figure 22), pulsed (Figure 25) and CW Doppler, as necessary. In these Type II patients, the high VSD velocity is indicative of subaortic obstruction. Interrogation of left ventricular outflow and PA region may reveal pulmonary or subpulmonary stenosis; the higher the velocity, the more severe the obstruction. Study from suprasternal notch may show aortic coarctation (Figure 26), which is not uncommon in patients with the Type II anatomy. 

A large number of other cardiac defects are known to be associated with tricuspid atresia [37,38]. Consequently, echo-Doppler studies should scrutinize for these defects during such studies.

## 7. Contrast Echocardiography in the Evaluation of Hypoxemia Following Open Heart Surgery

**Figure 27:**
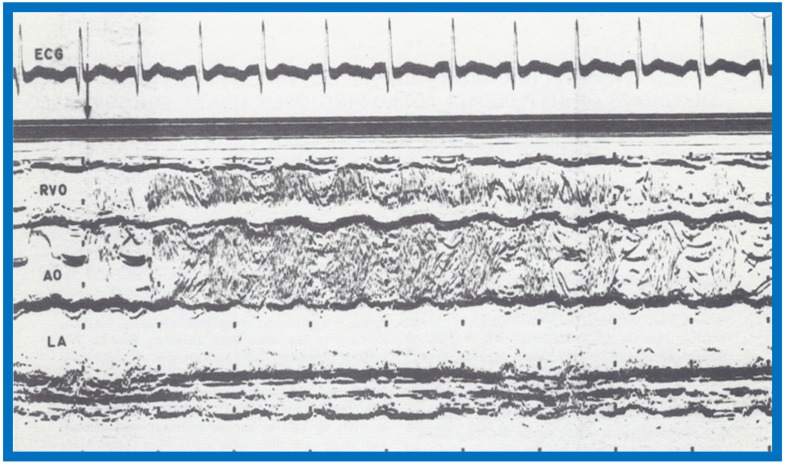
Selected M-mode recording from the parasternal short axis view of the left atrium (LA), aorta (Ao), and right ventricular outflow tract (RVO) while injecting agitated saline into the right atrial line, demonstrating the appearance of contrast echoes almost simultaneously in the Ao and RVO without opacification of the LA, and suggesting that there is no interatrial shunt, and that the shunt is distal to the atria. The time of injection is marked by an arrow at the left top of the figure. ECG, electrocardiogram. Reproduced from Rao P.S., et al. [8].

**Figure 28:**
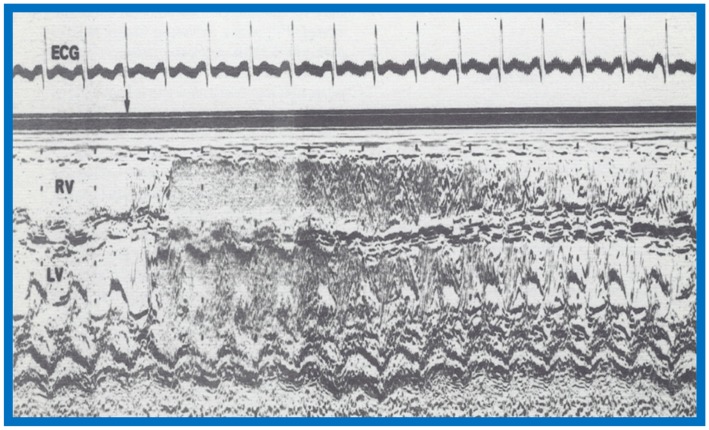
Selected M-mode recording from the parasternal short axis view of the left ventricle (LV) and right ventricle (RV) demonstrate the almost simultaneous appearance of contrast echoes in the LV and the RV, indicating that that the right-to-left shunt is at the ventricular level. The time of injection is marked by an arrow at the left top of the figure. ECG, electrocardiogram. Reproduced from Rao P.S., et al. [8].

We have used contrast echocardiography in other situations as well [8]. A three-year-old child had surgical correction of tetralogy of Fallot by patch closure of the VSD, resection of infundibular muscle and relief of valvar obstruction by a transannular pericardial patch. Following surgery, the child maintained good perfusion and normal vital signs, but had marked hypoxemia. Arterial PO_2_ was around 30 torr with O_2_ saturations in the mid 50s without any change in 100% O_2_. No cardiac murmurs were heard, and on the chest X-ray, no pulmonary pathology was identified. Since the foramen ovale was not closed during surgery, it was thought that patent foramen ovale (PFO) may be the reason for the hypoxemia. Contrast echocardiography was performed to confirm PFO as the site of right-to-left shunt. M-mode recordings from the parasternal short axis view of the LA, Ao, and right ventricular outflow tract (RVO) were made while injecting agitated saline into the right atrial line. This revealed the almost simultaneous appearance of contrast echoes in the Ao and RVO without opacification of the LA (Figure 27), suggesting that there was no interatrial shunt, and that the shunt was distal to the atria. A similar contrast study while recording the LV and right ventricle (RV) demonstrated the almost simultaneous appearance of contrast echoes in both the LV and the RV (Figure 28), indicating that that the right-to-left shunt was at the ventricular level. 

The patient was returned to the operating room and, upon inspection of the ventricular septum via right atriotomy, a second VSD was noted adjacent to previously closed VSD, separated by a muscle bundle. The defect was closed with another Dacron patch. The PFO was also closed with sutures. The PO_2_ and O_2_ saturation improved. Repeat contrast echo study did not reveal any evidence of right-to-left shunt. 

Other investigators used contrast echocardiography to demonstrate residual postoperative shunts, as reviewed in our paper [8], and our report further demonstrates the usefulness of this technique in evaluating postoperative residual shunts. We concluded that this technique is useful in identifying and localizing shunts in postoperative patients [8]. 

## 8. Quantitation of Left Ventricular Muscle Mass by M-Mode Echocardiography in Children

While there have been several studies on LV muscle mass (LVMM) by echocardiography in adult subjects, there had only been a limited number on echocardiographic measurements of the LVMM in children as of the early 1980s. The purpose of our investigation [9] was to establish normal echocardiographic LVMM in children, to identify sex- and race-related variation in LVMM, to establish if this measurement is sensitive to detect increased LVMM in children with sickle cell disease and to find the most appropriate method (from among the 3 available methods at that time) for calculating the LVMM in children. These data were presented in part at the VIII World Congress of Cardiology, Tokyo, Japan, in September 1978 [42].

We studied 204 normal children, aged 3-17 years; 107 were white and 97 black. The children were divided into 3 to 5, 6 to 10, 11 to 14 and 15 to 17 year age groups. The study subjects also included 47 children with sickle cell disease; 28 were boys and 19 were girls. All normal children were examined by one of the investigators; their normalcy was confirmed by physical examination and by ECG. Children with sickle cell disease had SS hemoglobin on hemoglobin electrophoresis, confirming their diagnoses. Weight, height, blood pressure, chest circumference and the antero-posterior diameter of the chest were measured in all children, as was their hemoglobin. Standard M-mode recordings from the parasternal short axis view of the left ventricle at the tips of the mitral valve were obtained, and measurements of LV end-diastolic (LVEDD) and end-systolic (LVESD) dimensions, as well as LV posterior wall and ventricular septal thickness in diastole and systole, were made in a standard fashion. The LVMM was calculated by three different methods, namely the cubed-function method and the methods described by Teichholtz and by Ratshin [9]. 

**Figure 29:**
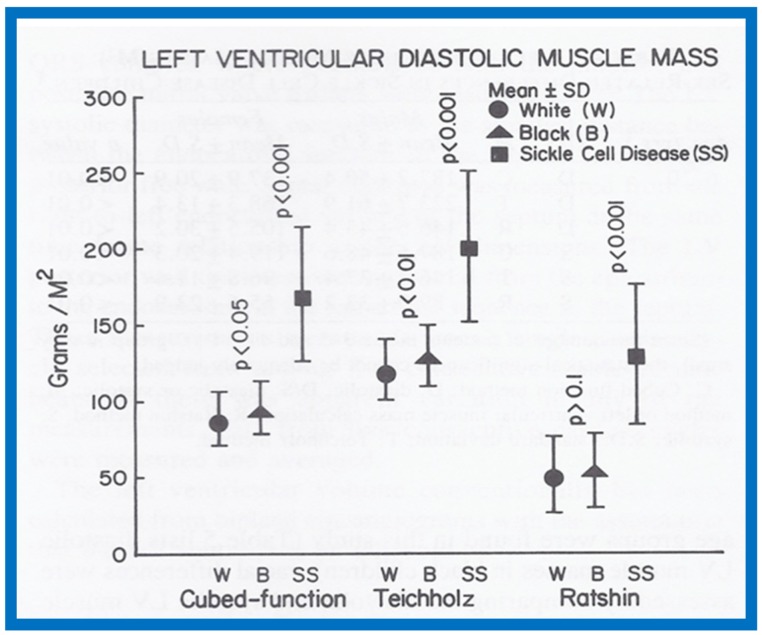
Left ventricular muscle mass in diastole expressed as g/M^2^ is illustrated for white (filled circles) and black (filled triangles) children and sickle cell disease patients (filled squares). Means and standard deviations are shown. Note higher muscle mass in black than in white children by cubed-function (*p* < 0.05) and Teichholz (*p* < 0.01) methods; this difference is not significant (*p* > 0.10 by Ratshin’s method. The diastolic left ventricular muscle mass in sickle cell disease is increased (*p* < 0.001) when compared with normal subjects by all three methods. Reproduced from Rao P.S., et al. [9].

**Figure 30:**
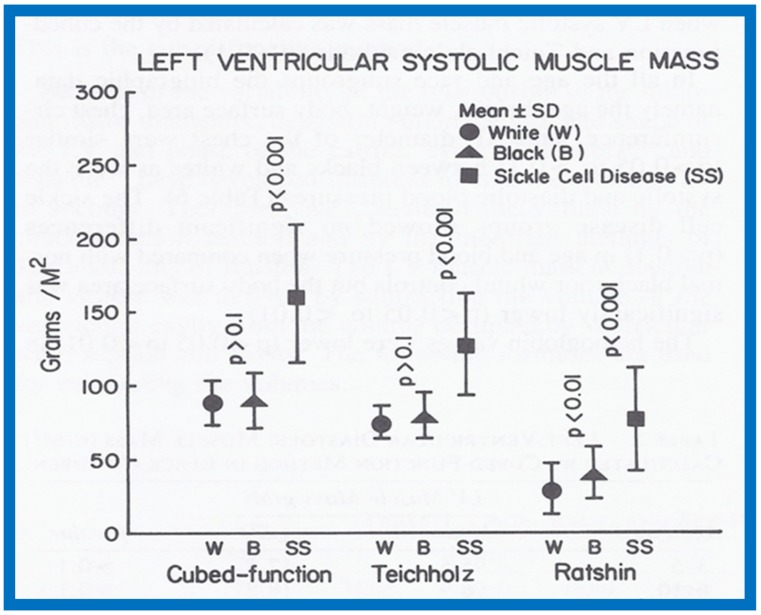
Left ventricular muscle mass in systole expressed as g/M^2^ is illustrated for white (filled circles) and black (filled triangles) children and sickle cell disease patients (filled squares). Means and standard deviations are shown. Note similar muscle mass in black and in white children by cubed-function (*p* > 0.1) and Teichholz (*p* > 0.1) methods and it is higher (*p* < 0.01) by Ratshin’s method. The systolic left ventricular muscle mass in sickle cell disease is increased (*p* < 0.001) when compared with normal subjects by all three methods. Reproduced from Rao P.S., et al. [9].

The results were presented in nine tables and three figures [9]. There were no gender-related differences in LVMM. There was also no significant difference in LVMM normalized to body surface area (g/M^2^) between the various age groups. Consequently, the LVMM data of all ages were combined, but were separated for the normal black and white children. The diastolic LVMM was higher (*p* < 0.05 to < 0.01) in black (96 ± 17 g/M^2^) than in white (89 ± 18 g/M^2^) children (Figure 29). Racial differences in systolic LVMM were less marked (Figure 30). The age distribution, weight, height, systolic and diastolic blood pressures, chest circumference and antero-posterior diameter of the chest were similar (*p* > 0.05 to *p* > 0.1) between black and white children, and were unlikely to have been responsible for LVMM differences. However, the hemoglobin was lower (*p* < 0.05 to < 0.01) in black than in white children, which may have explained their higher LVMM.

Both the diastolic (170 ± 44 g/M^2^) and systolic (163 ± 44 g/M^2^) LVMMs were higher (*p* < 0.001) in sickle cell disease children than those without (Figure 29 and Figure 30). The weight, height, body surface area, chest circumference and antero-posterior diameter of the chest and blood pressures were similar (*p* > 0.05 to > 0.1) in sickle cell disease vs. non sickle cell disease children. The causes for this increase may be related to chronic anemia producing augmented stroke volume and cardiac output with consequent LV hypertrophy similar to that seen thalassemia. The occlusion of coronary microcirculation by sickled red blood cells may be an additional factor. Among sickle cell disease children, the LVMM was larger (*p* < 0.05 to < 0.01) in boys than in girls. The reason for this is not clear, but may be related to differences in inappropriate response to anemia or incidence of sickling in the coronary circulation.

**Figure 31:**
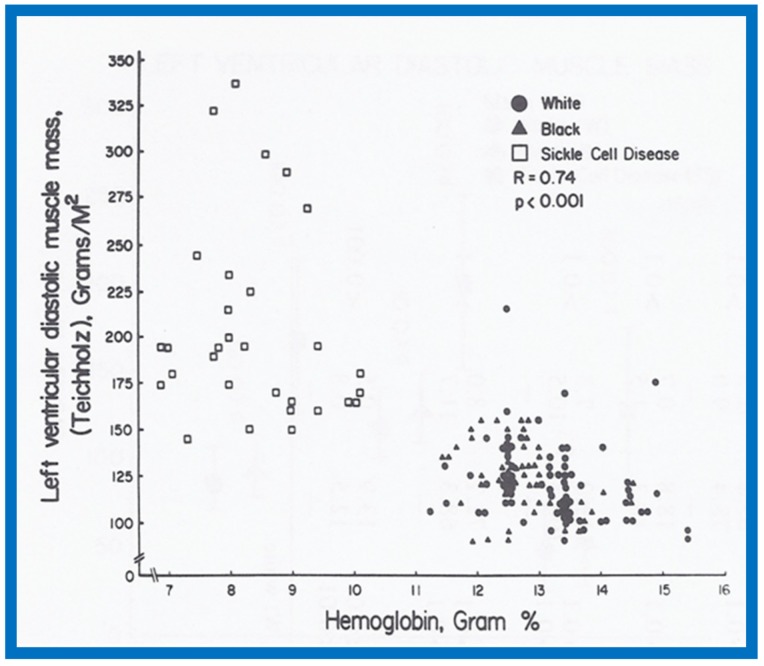
Left ventricular muscle mass in diastole calculated by Teichholz method is plotted against hemoglobin. Note significant (*R* = 0.74; *p* < 0.001) correlation between these parameters. Similar correlations were noted between the diastolic and systolic left ventricular muscle mass calculated by all three methods on the one hand, and hemoglobin values on the other. Reproduced from Rao P.S., et al. [9].

A significant correlation (*R* = 0.72, *p* < 0.001) was found between the level of hemoglobin and LVMM (Figure 31) for the entire group. 

Since multiple methods of measuring LVMM were available, we attempted to compare these methods to decide which was best for the evaluation of LVMM. All three methods were able to detect the increased LVMM in sickle cell patients. However, in the Ratshin’s method, the mean values were low with a wide scatter (white, 55 ± 25; black, 54 ± 24). In the method of Teichholz, there was a significant difference between the diastolic (white, 121 ± 20; black, 130 ± 20) and systolic (white, 75 ± 11; black, 77 ± 13) LVMM, while there was no such a difference in the cubed-function method (diastolic—white, 89 ± 18, black, 96 ± 17; systolic—white, 88 ± 15, black, 90 ± 19). In theory, there is no reason to have markedly different values for diastolic and systolic LVMM. For these reasons, we concluded that the cubed-function method may be better than the other two in the evaluation of LVMM in children [9]. The means and standard deviations for LVMM are listed in Table 2 [9]. 

On the basis of this study, it may be concluded that (i) there are no major gender-related differences in echocardiographic LVMM in children, (ii) the LVMM is larger in black than in white children, (iii) the LVMM in children with sickle cell disease is increased when compared with normal children, (iv) the cube-function method of LVMM calculation is better than the other two methods studied, and (v) normal LVMM in children is reasonably well established by this study (Table 2). 

## 9. Race and Sex Related Differences in Echocardiographic Measurements in Children

Although echocardiography was commonly used for the evaluation of infants and children with suspected heart disease in early 1980s, most of the normal standards did not take race or sex into consideration at that time. This was despite the fact that a substantial influence of gender and race on the ECGs had been shown. The purpose of our study was to scrutinize if sex and race had an important influence on conventional echocardiographic measures [10]. These data were presented in part at the first World Congress of Pediatric Cardiology, London, England, in June 1980 [43]. 

The study included a total of 273 black and white children of both sexes between the ages of 3 and 19; there were 136 black children and 137 white children and 143 were boys and 130 were girls. The subjects were divided into age groups of 3–5, 6–10, 11–14, and 15–19 years. As in our prior studies, all normal children were examined by one of the investigators, and their normalcy was confirmed by physical examination and by normal ECG. Echocardiographic data were also secured as previously detailed [5,6,9] (Figure 12 and Figure 13), and calculations were made as listed in Table 1. Weight, height, blood pressure (systolic and diastolic), chest circumference, antero-posterior diameter of the chest and hemoglobin were measured in all children. 

**Figure 32:**
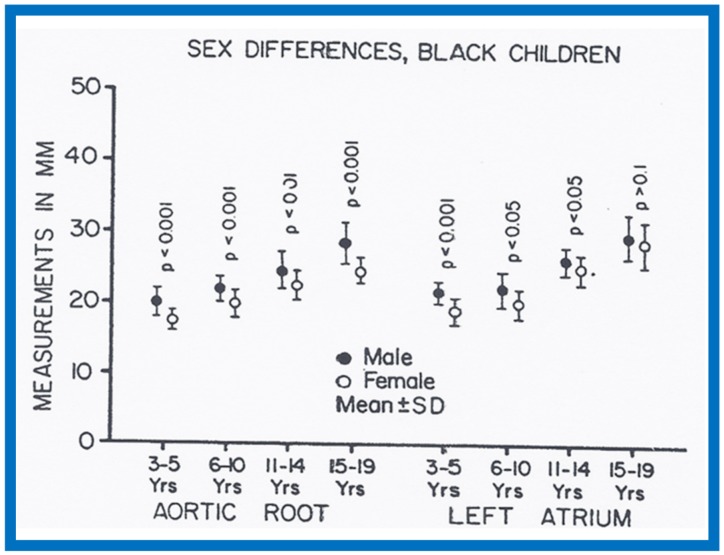
Gender-related differences in the measurements of aortic root and left atrium are illustrated for male black (filled circles) and female black (unfilled circles) children. Means and standard deviations (SD) are shown. Note larger (*p* < 0.001) aortic root size in males than females for all age groups. The left atrial size was larger (*p* < 0.05 to 0.001) in males than females in most age groups. Reproduced from Rao P.S., Thapar M.K. [10].

**Figure 33:**
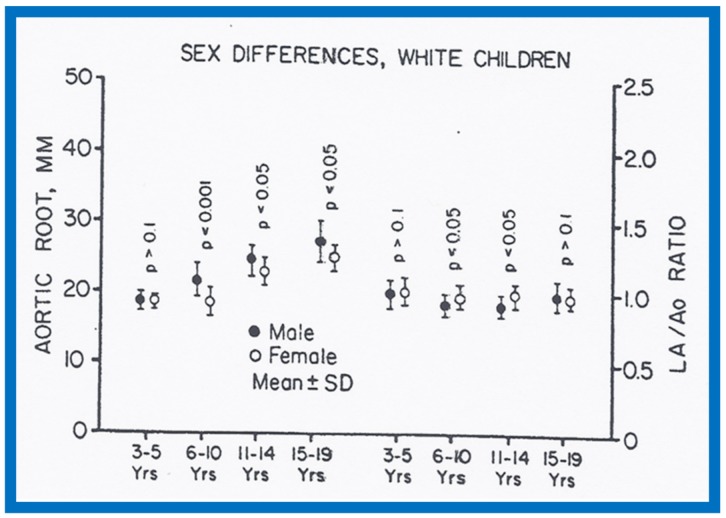
Gender-related differences in the measurements of aortic root and left atrium are illustrated for male white (filled circles) and female white (unfilled circles) children. Means and standard deviations (SD) are shown. Note that there is no consistent difference for all age groups in the aortic root and left atrial sizes between male and female white children. Reproduced from Rao P.S., Thapar M.K. [10].

There were no major gender-related differences (*p* > 0.05 to *p* > 0.1) in echocardiographic measurements with the exception of larger (*p* < 0.05 to *p* <0.01) Ao and LA in boys than in girls (Figure 32 and Figure 33). Larger Ao and LA is seen more consistently in black children than in white children. In addition, the LV posterior wall and interventricular septal thickness diastole were greater (*p* < 0.01) in teenage boys than girls. There were no differences (*p* > 0.05 to *p* > 0.1) in weight, height, blood pressure (systolic and diastolic), chest circumference, antero-posterior diameter of the chest and heart rate between boys and girls, but the hemoglobin was lower (*p* < 0.05 to *p* <0.01) in 15–19-year-old girls than boys. The reason for larger Ao in boys than girls remains unexplained.

**Figure 34:**
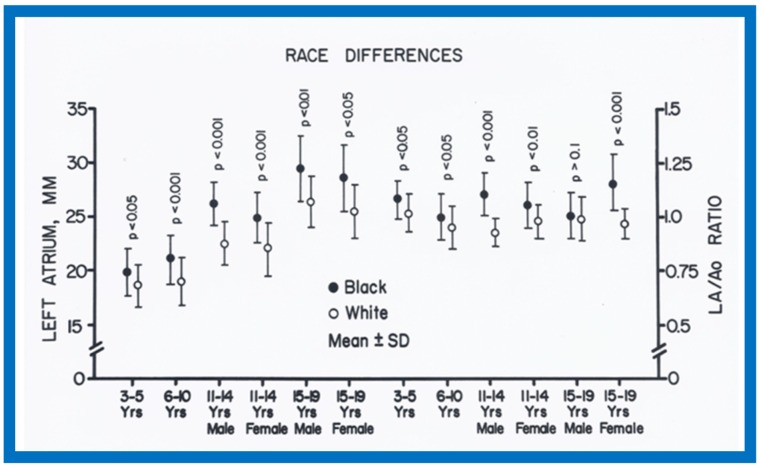
Race-related differences in the measurements of left atrium (LA) and LA to aortic root (Ao) ratio are illustrated for black (filled circles) and white (unfilled circles) children. Means and standard deviations (SD) are shown. Note the larger (*p* < 0.05 to < 0.001) LA and LA/Ao ratio in black than white children for all age groups. Reproduced from Rao P.S., Thapar M.K. [10].

**Figure 35:**
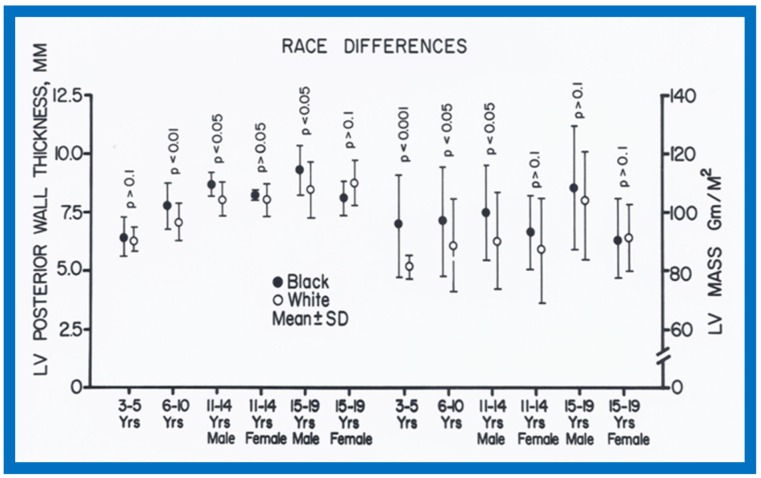
Race-related differences in the measurements of left ventricular (LV) posterior wall thickness and LV mass are illustrated for black (filled circles) and white (unfilled circles) children. Means and standard deviations (SD) are shown. Note the larger (*p* < 0.05 to < 0.001) LV posterior wall thickness and LV mass in black than white in all but 11-19 year-old females. Reproduced from Rao P.S., Thapar M.K. [10].

**Figure 36:**
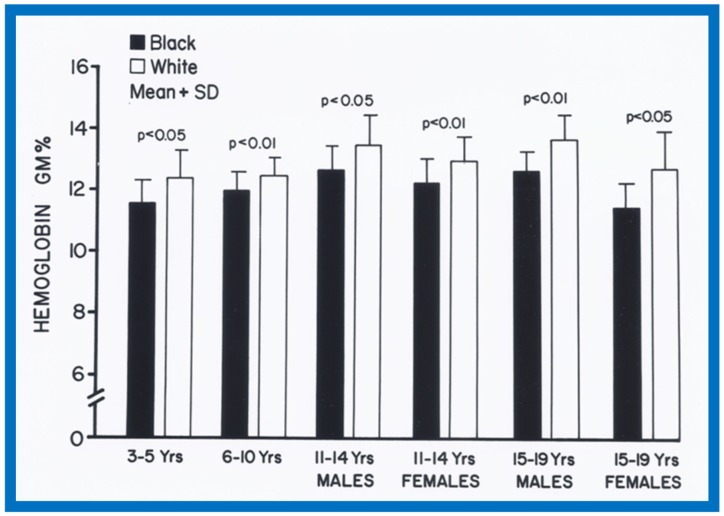
Bar graph showing comparison of hemoglobin, gm% in black (filled bars) and white (open bars) white children. Means and standard deviations (SD) are shown. Note the significantly lower (*p* < 0.05 to < 0.01) hemoglobin values in black than white children for all age-gender subgroups. Reproduced from Rao P.S., Thapar M.K. [10].

When racial differences were examined, larger (*p* < 0.05 to 0.0001) LA and/or LA/Ao ratio in black than white children of all age-sex subgroups were found (Figure 34). Also, a thicker LV posterior wall and/or LV muscle mass (in black than in white) was found in most age–gender subgroups (Figure 35). The weight, height, body surface area, chest circumference, antero-posterior diameter of the chest and blood pressure were similar (*p* > 0.05), but lower (*p* < 0.05 to 0.01) hemoglobin levels in black than white children were seen (Figure 36) and may, in part, be responsible for the echo differences.

In contrast to significant gender- and race-related ECG differences, the echo differences are few. Normal echocardiographic tables incorporating the detected differences along with percentile distributions are listed in multiple tables in the appendix of our publication [10]. 

## 10. Cardiac Size and Function in Patients with Sickle Cell Disease

One hundred and twenty-four children with sickle cell anemia and 78 healthy black children (controls) were studied by echocardiography to evaluate cardiac size and function in sickle cell patients [11]. A progressive increase in LV size and left ventricular muscle mass was observed with increasing age in sickle cell patients, while no such changes were seen in control subjects. The LV shortening fraction and Vcf increased in sickle cell patients, indicating normal LV systolic function by these measures. However, the pre-ejection period (*p* < 0.05) and the pre-ejection to ejection time ratio (*p* < 0.005) were greater in sickle cell patients than in controls. Systolic time intervals became increasingly abnormal in the sickle cell subjects with increasing age. We conclude that sickle cell patients develop progressive increases in LV size and mass, as well as progressive deterioration of LV function with age [11].

## 11. Afterload Reduction in the Management of Primary Myocardial Disease

While the use of inotropic agents and diuretics in the management of heart failure in children is widely accepted, the use of afterload reduction had not been well recognized in the management of children with heart failure as of early and mid-1980s. Therefore, we sought to explore the afterload reduction principle in the management of infants and children with primary myocardial disease (PMD) [12]. The results of this study were presented in part at the 2nd World Congress of Pediatric Cardiology in June 1985 [44] and at the at the 54th Annual Scientific Meeting of the American Academy of Pediatrics in October 1986 [45]. 

Cardiac catheterization and selective cine-angiography confirmed the clinical diagnosis of PMD in all but one patient. This lone exception child had anomalous origin of the left coronary artery from the pulmonary artery and was excluded from the study. Twenty-six infants and children, aged 3 to 48 months with PMD, were divided into two groups: the first group received conventional anticongestive therapy including digitalis and diuretics while the second group, in addition to conventional therapy, also received hydralazine (4.0 mg/kg/day in four divided doses). In the hydralazine group, the administration of intravenous hydralazine (0.2–0.25 mg/kg) during cardiac catheterization resulted in increased cardiac index (3.18 ± 0.29 vs. 3.75 ± 0.39 L/min/M^2^; *p* < 0.01) and decreased systemic vascular resistance (18.68 ± 2.47 vs. 15.4 ± 2.28 units; *p* < 0.05) without any change in mean arterial pressure (63.6 ± 9.93 vs. 63.7 ± 8.27 mmHg; *p* > 0.1). Echocardiographic studies were initially performed at 1- to 3-month intervals, and later, at 6-month intervals. 

**Figure 37:**
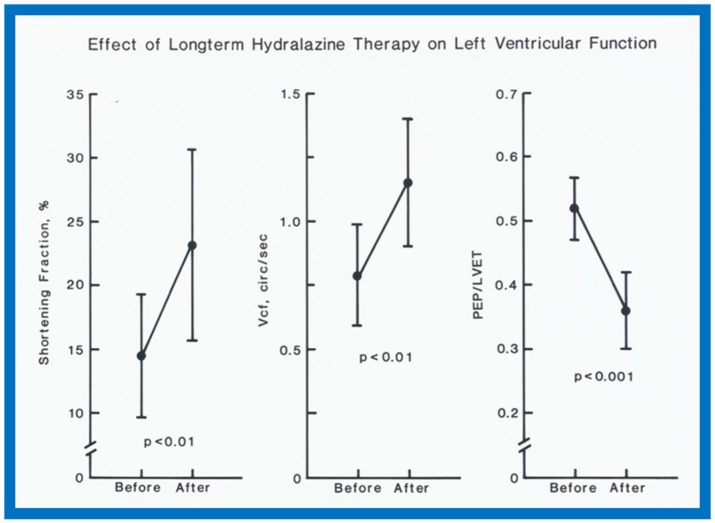
Left ventricular (LV) function indices, namely shortening fraction in percent (%) (left panel), velocity of circumferential fiber shortening (Vcf) in circumferences/sec (center panel) and pre-ejection period (PEP)/LV ejection time (LVET) ratio (right panel) prior to (Before) and following (After) hydralazine therapy are illustrated. Means and standard deviations are shown. Note the significant (*p* < 0.01 to 0.001) improvement in all LV function indices. Circ: circumferences.

**Figure 38:**
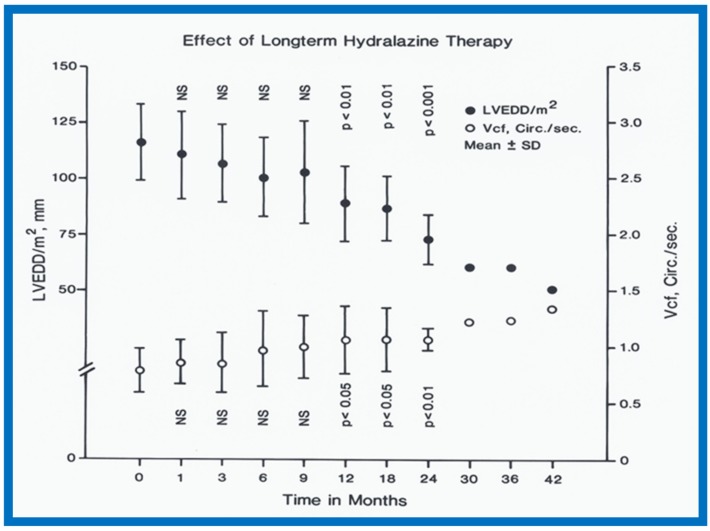
Left ventricular end-diastolic dimension (LVEDD)/m^2^ in mm (closed circles) and velocity of circumferential fiber shortening (Vcf) in circumferences/sec (open circles) are shown from prior to start of hydralazine therapy (0) and at 1, 3, 6, 9, 12, 18, 24, 30, 36 and 42 months following initiation of hydralazine therapy. Means and standard deviations (SD) are shown. The number of subjects at 30, 36 and 42 months is small, and therefore, only mean values are shown. Note the gradual improvement in LVEDD and Vcf. Statistically significant (*p* < 0.05 to < 0.01) change becomes apparent from 12 months follow-up onwards. Reproduced from Rao P.S., Andaya W.G. [12].

**Figure 39:**
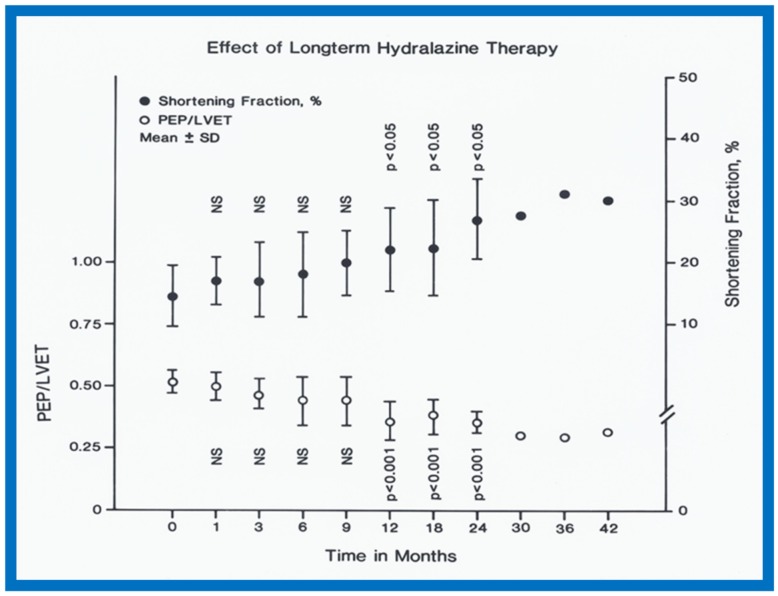
Pre-ejection period (PEP)/left ventricular ejection time (LVET) ratio (open circles) and shortening fraction in percent (%) (closed circles) are shown from prior to start of hydralazine therapy (0) and at 1, 3, 6, 9, 12, 18, 24, 30, 36 and 42 months following initiation of hydralazine therapy. Means and standard deviations (SD) are shown. The number of subjects at 30, 36 and 42 months is small and therefore, only mean values are shown. Note the gradual improvement in PEP/LVET and shortening fraction. Statistically significant (*p* < 0.05 to < 0.001) change becomes apparent from 12 months follow-up onwards. Reproduced from Rao P.S., Andaya W.G. [12].

In the hydralazine group, at a median follow-up of 21 months, the LV dimension decreased; (116 ± 7 vs. 87 ± 21 mm/M^2^; *p* < 0.01), shortening fraction increased (14.5 ± 4.9 vs. 23.2 ± 7.5; *p* < 0.01) and Vcf (0.79 ± 0.2 vs. 1.15 ± 0.55 circ/sec; *p* < 0.01) (Figure 37), and the ratio of pre-ejection period to LV ejection time normalized (0.52 ± 0.05 vs. 0.35 ± 0.06; *p* < 0.001) (Figure 37). These improvements occurred gradually but became statistically significant (*p* < 0.05 to < 0,001) at 12 months following the initiation of hydralazine therapy (Figure 38 and Figure 39). Other indices of LV function also improved, as detailed in the Table from our paper [12] for the interested reader. 

**Figure 40:**
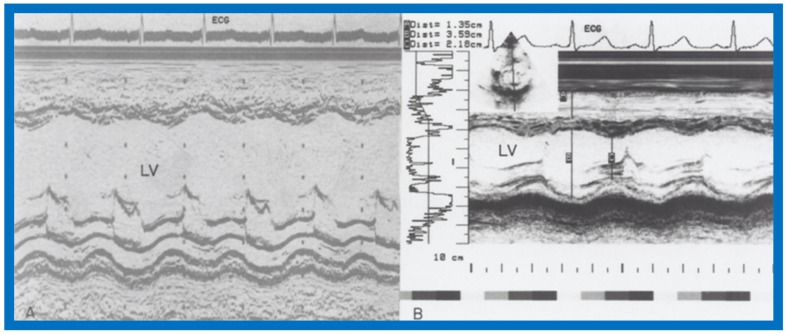
Selected M-mode recording from the parasternal short axis view of the left ventricle (LV) prior to (**A**) and following (**B**) hydrazine therapy. Note the significant improvement in the LV size and function.

**Figure 41:**
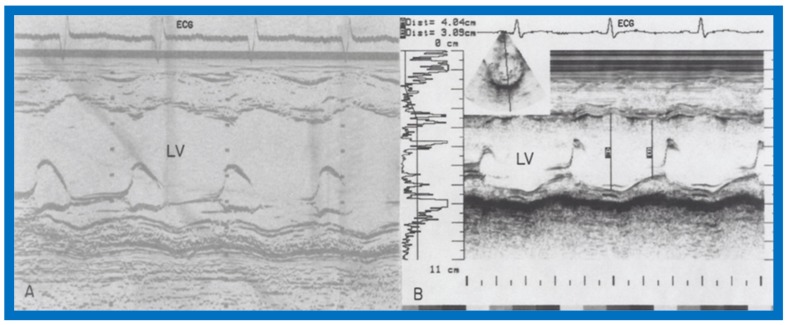
Selected M-mode recording from the parasternal short axis view of the left ventricle (LV) prior to (**A**) and following (**B**) hydrazine therapy from a different infant. Also, note the significant improvement in the LV size and function.

**Figure 42:**
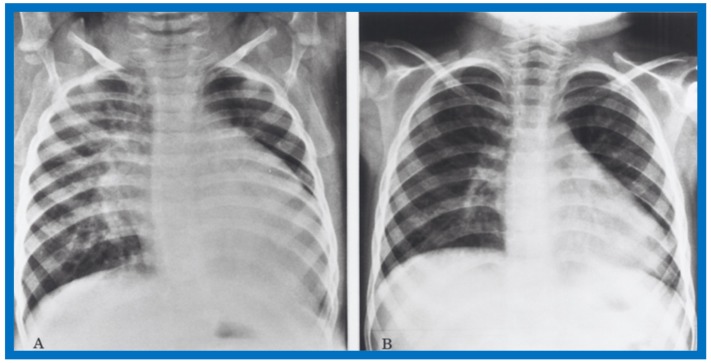
Antero-posterior view of chest x-rays prior to (**A**) and following (**B**) hydralazine therapy. Moderate cardiomegaly and pulmonary venous congestion were seen prior to therapy (**A**) which improved remarkably after therapy (**B**).

**Figure 43:**
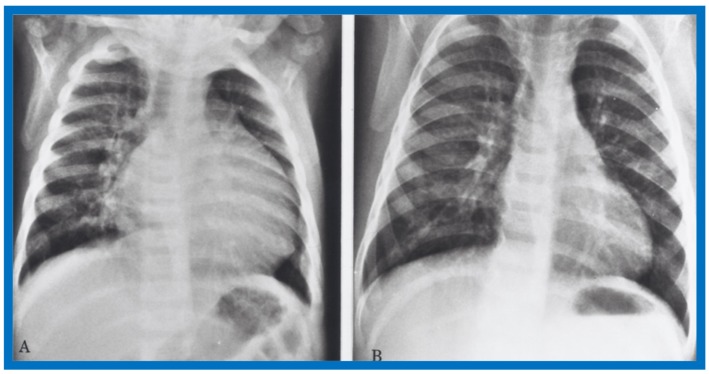
Antero-posterior view of chest x-rays prior to (**A**) and following (**B**) hydralazine therapy from a different infant. Moderate cardiomegaly with pulmonary venous congestion was seen prior to therapy (**A**) which improved remarkably after therapy (**B**), similar to that seen in Figure 42.

Examples of improvement in LV dimension on echo studies are shown in Figure 40 and Figure 41. The cardiac size and pulmonary venous congestion on chest roentgenograms improved in the hydralazine group (Figure 42 and Figure 43). No significant changes in any of these parameters occurred in the control group. 

While this was a prospective study, we did not use concurrent controls, nor was this a double-blind randomized study with placebo administration in the control group. Consequently, we suggested a double-blind randomized study to confirm our findings. We stated that hydralazine does not alter the basic cardiac disease, but by improving the cardiac output, prolongs the patients’ life, giving an opportunity for recovery of cardiac function and spontaneous improvement, should that be the natural history of disease. We concluded that hydralazine therapy is a valuable addition in the management of PMD in infants and children [12]. Subsequent to the initiation of our study, afterload reducing, angiotensin-converting enzyme (ACE) inhibitors came into vogue and may be considered superior to hydralazine. 

In a chapter in Current Therapy [46] and in an editorial [47] published during the early part of the above study, the author reviewed the concept of afterload reduction. The use of intravenous vasodilators (sodium nitroprusside and nitroglycerin) to treat postoperative patients with low cardiac output and oral vasodilators (prazosin and hydralazine) to treat chronic congestive heart failure were also reviewed [46,47]. Indeed, these reviews, in part, enticed the author to initiate the afterload reduction study [12] just reviewed.

In an editorial communication in 1995 [48], we commented on the use of ACE inhibitors instead of vasodilators such as hydralazine as afterload reducing agents to treat dilated cardiomyopathy.

## 12. Echo-Doppler Studies in the Evaluation of the Results of Balloon Pulmonary Valvuloplasty

**Figure 44:**
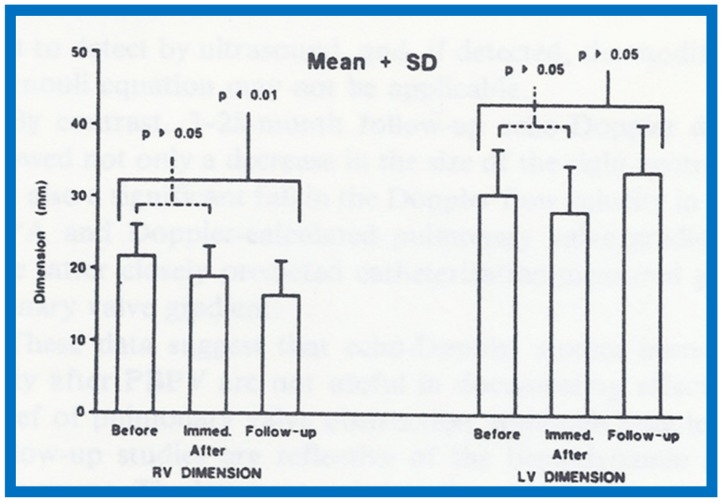
Bar graph showing right (RV) and left (LV) ventricular end-diastolic dimensions prior to (Before), immediately after (Immed. After) balloon pulmonary valvuloplasty and at a median follow-up of 14 months (Follow-up). Means and standard deviations (SD) are shown. Note that there is small but not statistically significant (*p* > 0.05) decrease in RV dimension immediately after balloon pulmonary valvuloplasty, but at follow-up, there was a significant (*p* < 0.01) decrease in RV dimension. There was no change (*p* > 0.05) in LV dimension either immediately after balloon pulmonary valvuloplasty or at follow-up. Reproduced from Rao P.S. [13].

**Figure 45:**
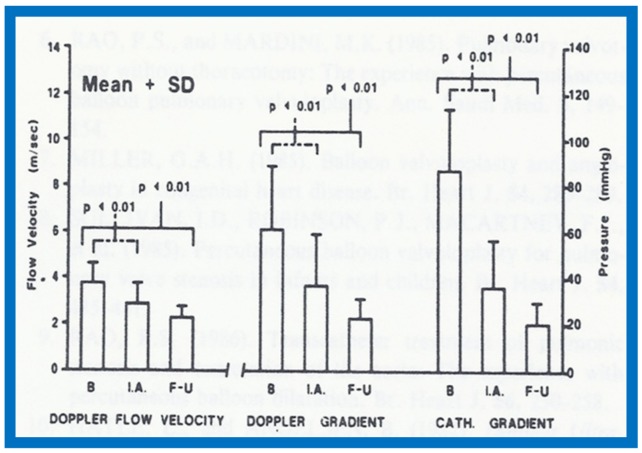
Bar graph showing Doppler flow velocity (left panel) in m/sec, calculated Doppler gradient (center panel) in mmHg and catheterization (CATH) measured peak-to-peak gradient (right panel) in mmHg before (B), immediately after (I.A.) and at a median follow up of 14 months (F-U) after balloon pulmonary valvuloplasty. Means and standard deviations (SD) are shown. Note that there is significant (*p* < 0.01) decrease in Doppler flow velocity, calculated Doppler gradient and catheterization measured gradient both immediately after balloon pulmonary valvuloplasty and at follow-up. Reproduced from Rao P.S. [13].

The utility of echo-Doppler studies in the assessment of the results of balloon dilatation of the pulmonary valve was examined [13]. While the right ventricular end-diastolic dimension (RVED) did not significantly change (*p* > 0.05) immediately following balloon pulmonary valvuloplasty (Figure 44), the Doppler flow velocity and calculated pressure gradient across the pulmonary valve (Figure 45) decreased significantly (*p* < 0.01). 

However, there was some discrepancy between the Doppler-derived and catheterization-measured transpulmonary valve gradients in four patients. These patients had either severe stenosis (catheterization gradients between 94 to 190 mmHg) or severe residual infundibular stenosis immediately following balloon valvuloplasty. In one patient, despite a fall in pulmonary valvar gradient by catheterization, the Doppler flow velocity did not decrease. We explored potential causes for this discrepancy and excluded technical aspects of Doppler recording, but surmised that small vertex shed distance in small orifices or cone-shaped spray formation instead of in a focused jet, making it difficult to detect by ultrasound, as postulated by Vasko and associates [49], which may explain the discrepancy. Or, as our later experience suggested, this may in part be related to RV infundibular constriction which occurred immediately after balloon pulmonary valvuloplasty.

**Figure 46:**
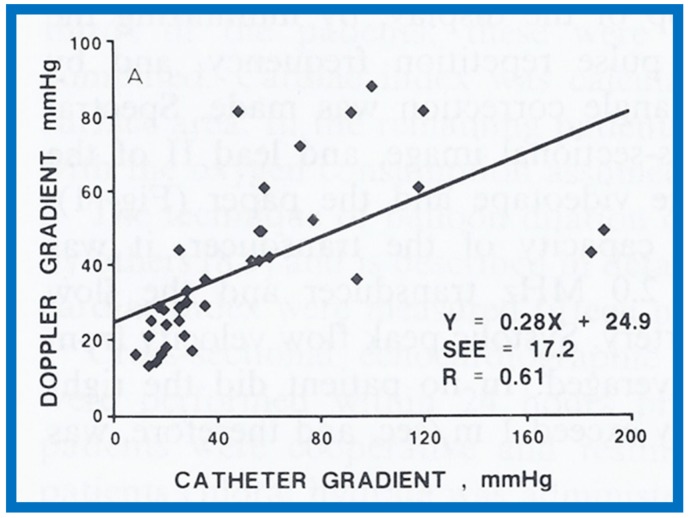
Scattergram demonstrating the relationship of Doppler-derived (by modified Bernoulli equation) peak instantaneous and catheterization-measured peak to peak pulmonary valve systolic pressure gradients. Note that the linear regression analysis indicated a correlation coefficient (R) of 0.61. Reproduced from Rao P.S. [14].

**Figure 47:**
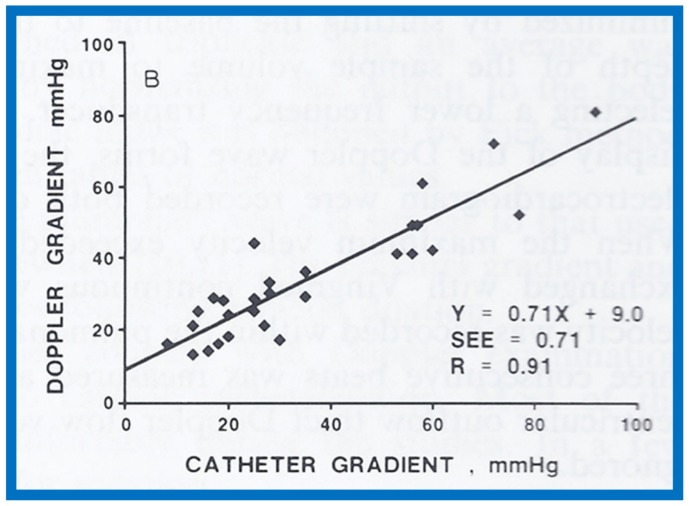
Scattergram demonstrating the relationship of Doppler-derived (by modified Bernoulli equation) peak instantaneous and catheterization-measured peak to peak pulmonary valve systolic pressure gradients; this is similar to Figure 33, but after removal of data sets form five patients with severe stenosis and one patient with severe infundibular stenosis. Note that the linear regression analysis indicated improvement in correlation coefficient (R) to 0.91. Reproduced from Rao P.S. [14].

At follow-up (3 to 28 months after valvuloplasty), the RVED (Figure 46) decreased significantly (*p* < 0.01), as did the Doppler flow velocity and calculated pressure gradient across the pulmonary valve (Figure 47) (*p* < 0.01). The Doppler gradient reduction is similar to that obtained by cardiac catheterization concurrently. In addition, there was a good correlation (*r* = 0.8) between Doppler-derived and catheterization-measured transpulmonary valve gradients at follow-up. 

It was concluded that echo-Doppler studies may not be truly indicative of the immediate results, but are reflective of the results at follow-up; the latter may obviate the need for repeat cardiac catheterization for evaluations of follow-up results of balloon pulmonary valvuloplasty [13].

## 13. Doppler in the Prediction of Pressure Gradients in Valvar Pulmonary Stenosis 

**Figure 48:**
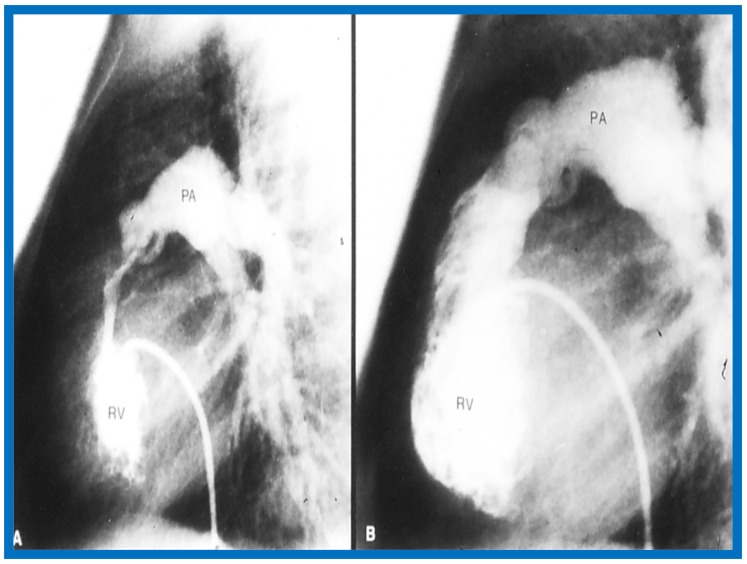
Selected right ventricular (RV) cineangiographic frames from lateral projection demonstrating severe infundibular constriction immediately following balloon pulmonary valvuloplasty (**A**) which has resolved (**B**) during a study six months later. PA, pulmonary artery. Reproduced from Rao P.S. [14].

**Figure 49:**
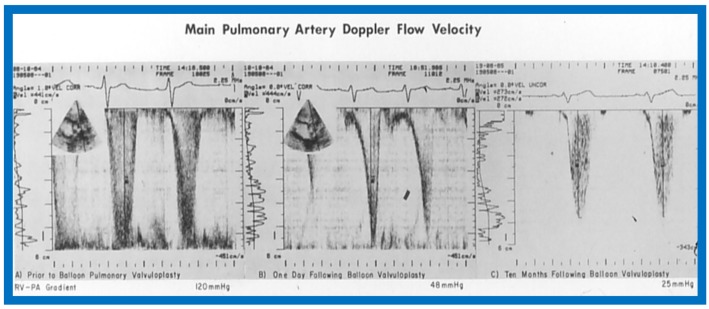
Doppler flow velocity recordings from the main pulmonary artery prior to (**A**), immediately after (**B**) balloon pulmonary valvuloplasty and at 10-month follow-up (**C**). Note the high Doppler flow velocity prior to balloon pulmonary valvuloplasty indicating severe gradient in A; the Doppler velocity immediately after balloon pulmonary valvuloplasty has a triangular pattern, indicating the infundibular gradient in B and corresponding to A in Figure 50. At follow-up 10 months later, the Doppler velocity had fallen (**C**) and corresponded to B in Figure 50, suggesting resolution of infundibular obstruction. Reproduced from Rao P.S. [14].

The relationship of Doppler with catheterization-measured pulmonary valve pressure gradients was studied by examining 35 pairs of such measurements, made within 24 h of each other [14]. The correlation coefficient was 0.61 (Figure 46); this improved to 0.91 (Figure 47) after removal of the five patients with severe stenosis (gradients of 94 to 190 mmHg) and one patient with severe infundibular stenosis (Figure 48 and Figure 49). 

It was postulated that a cone-shaped spray instead of a focused jet in subjects with extremely severe pulmonary valve stenosis may the be reason for Doppler’s inability to predict the gradient measured at cardiac catheterization [13,50]. It was concluded that Doppler is good tool to estimate transvalvar gradients in pulmonary stenosis, provided extremely severe valvar stenosis and infundibular stenosis patients are excluded.

## 14. Echocardiographic Demonstration of Atrioventricular Type of Tricuspid Atresia

**Figure 50:**
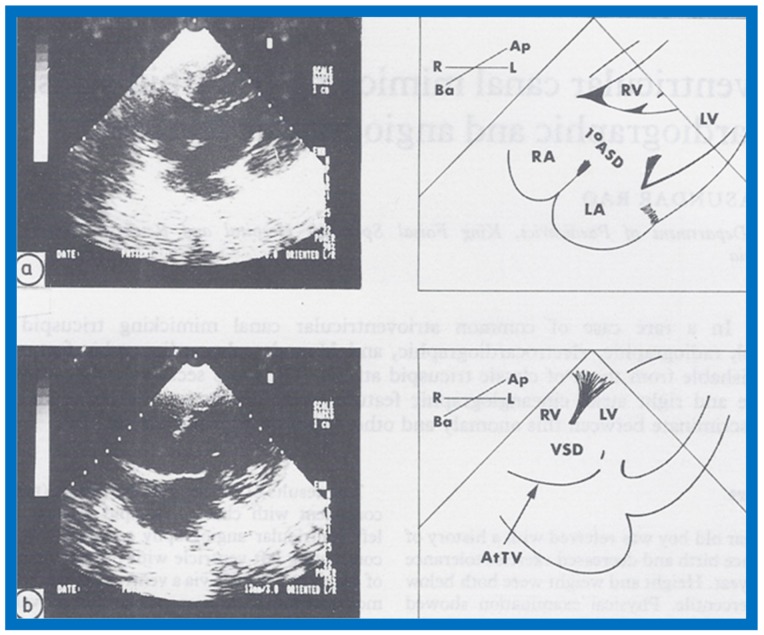
Selected two-dimensional, subcostal, four-chamber echocardiographic frames with an open (**a**) and closed (**b**) atrioventricular valve. Line drawings on the right of **a** and **b** are made for greater clarity and for labeling. A large ostium primum atrial septal defect (1^0^ ASD) is shown in a. When the large atrioventricular valve leaflet is open (**a**), it completely closes the right ventricle (RV) from the right atrium (RA) and ventricular septal defect (VSD) and allows emptying of blood from both atria into the left ventricle (LV). When atrioventricular valve leaflet is closed (**b**), it continues to occlude the RV from the RA while allowing the VSD to freely communicate between RV and LV. Ap, Apex; AtTV, atretic tricuspid valve; Ba, base; L, left; LA, left atrium; R, right. Reproduced from Rao P.S. [15].

**Figure 51:**
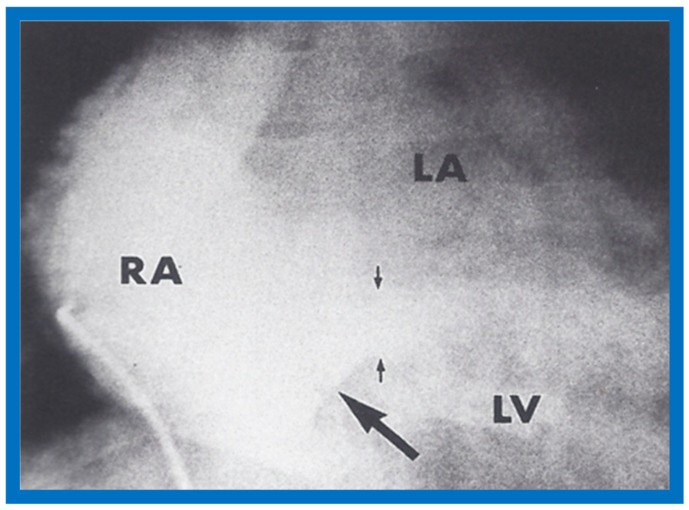
Selected right atrial (RA) angiographic frame in postero-anterior view demonstrating that the floor of the right atrium is formed by one of the leaflets of the atrioventricular valve; this is marked by a large arrow. The contrast material exited the RA via an ostium primum atrial septal defect shown by small arrows with subsequent opacification of the left ventricle (LV). C, catheter; LA, left atrium. Reproduced from Rao P.S. [15].

The author reported on the clinical, radiographic, ECG, echocardiographic and hemodynamic features of a rare case of common atrioventricular canal (currently called atrioventricular septal defect) mimicking tricuspid atresia in an eight-year-old child [15]. The clinical, radiographic and ECG characteristics were similar to those of classic tricuspid atresia. Hemodynamic data secured at cardiac catheterization were also similar to those seen with tricuspid atresia. A two-dimensional echocardiogram demonstrated an ostium primum atrial septal defect with a common atrioventricular valve and a small RV (Figure 50a,b); the entry into the RV appeared to be occluded by a leaflet of the common atrioventricular valve. Left ventricular cineangiogram in postero-anterior view demonstrated “gooseneck” deformity, similar to that seen in atrioventricular canal defects [15]. However, right atrial angiography resulted in direct emptying of the contrast material into the left ventricle via an ostium primum atrial septal defect (Figure 51). The floor of the right atrium seemed to be formed by a leaflet of the common atrioventricular valve (Figure 51). Thus, the two-dimensional echocardiographic and right atrial cineangiographic features appeared to be distinctive, and could be utilized to distinguish this anomaly from other varieties of tricuspid atresia.

**Figure 52:**
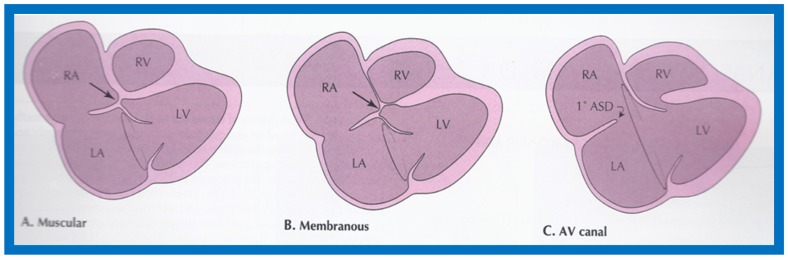
Line drawings demonstrating two dimensional echocardiographic appearances in subcostal four-chamber view of the muscular (**a**), membranous (**b**), and atrioventricular canal (**c**) variants of tricuspid atresia. (**a**) The atretic tricuspid valve is represented by a thick band of echoes between the right atrium (RA) and the small right ventricle (RV) in the muscular type. (**b**) The tricuspid valve is represented by a thin line in the membranous type. Note that crux of the heart (arrows in **a** and **b**) is well seen in both these types (**a** and **b**). The attachment of the anterior leaflet of the detectable atrioventricular valve to the left side of the interatrial septum is evident. (**c**) In the atrioventricular canal type of tricuspid atresia, the anterior leaflet of the detectable atrioventricular canal is attached to the anterior wall of the heart, occluding the right ventricle from the right atrium and allowing blood exit of both atria into the left ventricle (LV). Crux cordis and the atrioventricular portion of the interventricular septum are not seen. Reproduced from Rao P.S. [15,40].

The reported anomaly is definitely different from classic tricuspid atresia, but has physiologic effects similar to classic tricuspid atresia. A detailed review of the literature at that time suggested that this anomaly is extremely rare, with only one brief mention of a similar case by Van Praagh by that time [50]. Evaluation of crux cordis (Figure 54) on two-dimensional echocardiogram (subcostal four-chamber view) may help to distinguish these anomalies from each other. In muscular type of tricuspid atresia, a dense band of echoes is seen where the normal tricuspid valve should be (Figure 52a). In membranous types of tricuspid atresia, a thin membrane is seen instead (Figure 52b). In both types, the anterior leaflet of the detectable atrioventricular valve is attached to the left side of interatrial septum (Figure 52a,b). In the atrioventricular canal type of tricuspid atresia, the crux cordis is abnormal and cannot be identified, and a large atrioventricular valve leaflet occludes the entry of the RA into the RV (Figure 52c). It was concluded that two-dimensional echocardiographic and angiographic features help differentiate atrioventricular canal type of tricuspid atresia from classic tricuspid atresia cases [15].

## 15. Review: Doppler Echocardiography in Noninvasive Diagnosis of Heart Disease

The author reviewed the role of Doppler echocardiography in noninvasive diagnoses of heart disease in infants and children in the late 1980s [16]. The purpose of that review was to describe the principles of Doppler echocardiography and its clinical applications. Doppler is the study of low magnitude echoes from blood cells which are rejected in regular, imaging echocardiography. Doppler, by using modified Bernoulli equation and measured arm blood pressure, along with some assumptions, facilitates in providing flow and pressure measurements from the heart chambers and the blood vessels. Thus, Doppler supplements the anatomic information provided by the imaging echocardiography, and should be performed in conjunction with regular echocardiographic study. 

The discussion began by explaining ultrasound, its transmission into the body producing echoes, and the rejection of low intensity echoes from blood cells in conventional imaging echocardiography, which were studied by applying the principle of Doppler (apparent shift of transmitted frequency by moving blood cells) and analysis of frequency shift by Fourier transformation or Chrisp-Z methodology built into the echo-Doppler equipment. There are many types of Doppler, including pulsed Doppler, continuous wave Doppler, high pulsed rate frequency Doppler and color Doppler. Discussion of color flow Doppler imaging was not included in that review. Each type of Doppler has its own advantages and disadvantages. This is followed by a discussion of how Doppler data is acquired. The clinical application of Doppler to include calculation of cardiac output/index, qualitative and quantitative estimation of intracardiac shunts (atrial and ventricular septal defect and patent ductus arteriosus), the detection and quantification of semilunar and atrioventricular valve stenosis and insufficiency were briefly discussed, with examples of Doppler images for most of the lesions included in the discussion. It was concluded that Doppler studies should be performed in conjunction with imaging echocardiography, and that it is a useful adjunct to it [16]. 

## 16. Echo-Doppler Studies in the Evaluation of the Results of Balloon Angioplasty of Aortic Coarctation

**Figure 53:**
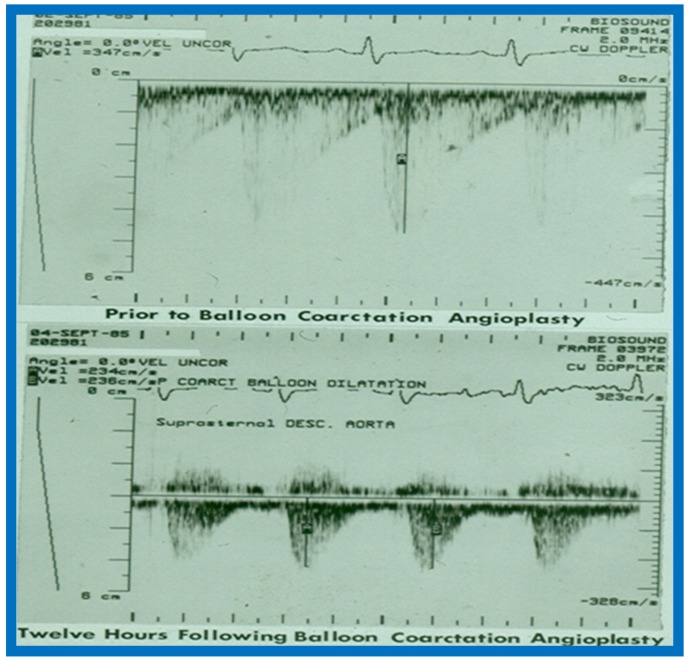
Continuous wave Doppler flow velocity recordings from suprasternal notch view directing the Doppler signal towards the descending aorta prior to (top) and twelve hours following (bottom) balloon angioplasty of aortic coarctation. Note the reduction of peak Doppler flow velocity from 3.47 m/s to 2.35 m/s after balloon angioplasty. Also note that the pandiastolic flow seen prior to angioplasty is no longer seen after angioplasty. Reproduced from Rao P.S. [17].

**Figure 54:**
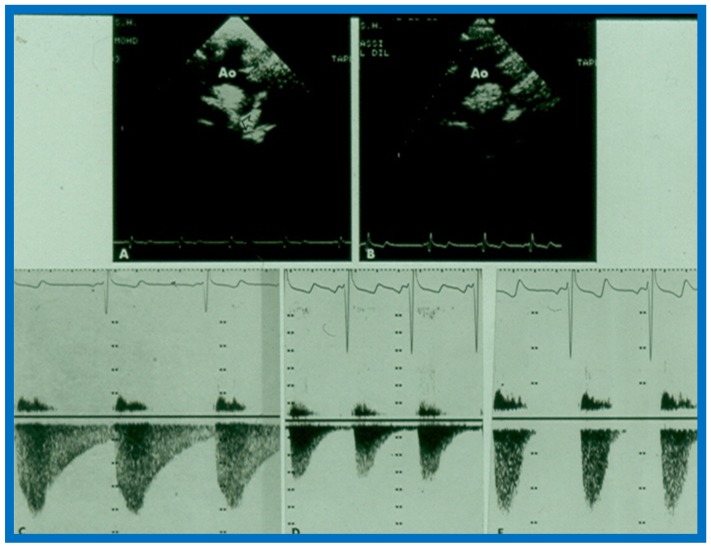
(**A**,**B**) Two-dimensional (2D) echo images prior to (**A**) and following (**B**) balloon angioplasty of aortic coarctation show improvement in (**B**). (**C**–**E**) Continuous wave Doppler flow velocity recordings from suprasternal notch directing the Doppler signal towards the descending aorta prior to (**C**) and immediately following (**D**) balloon angioplasty of aortic coarctation and at six months after angioplasty (**E**) are shown. Note the reduction of peak Doppler flow velocity from (**C**) to (**D**) with further fall in (**E**). Also note that the diastolic flow is seen throughout the entire diastole (pandiastolic) prior to angioplasty (**C**), and is seen only is early diastole immediately after angioplasty (**D**) and at six-month follow-up (**E**), there was no diastolic flow at all. Reproduced from Rao P.S. [17].

This study sought to appraise the usefulness of echo-Doppler studies in evaluating immediate and follow-up results of balloon angioplasty of native aortic coarctation [17]. Echo-Doppler data prior to and immediately following angioplasty were available for 19 infants and children, and such data at follow-up (3 to 22 months; mean 12 months) were accessible for review in 18 patients. There was no significant change (*p* > 0.1) in left ventricular (LV) end-diastolic dimension, LV posterior wall thickness in diastole and LV shortening fraction either immediately after balloon angioplasty or at follow-up. However, the Doppler flow velocity (3.2 ± 0.85 vs. 2.5 ± 0.5 m/s) and the calculated pressure gradients (∆P = 4V^2^) across the coarctation 43.3 ± 21.9 vs. 26.5 ± 10.3 mmHg) decreased immediately after (*p* < 0.01) balloon angioplasty, as well as at follow-up (*p* < 0.02). But there was not a good correlation between the Doppler derived and catheterization-measured gradients (*r* = 0.49–0.78). It was also observed that pandiastolic anterograde descending aortic flow shortened/disappeared (Figure 53 and Figure 54) in all patients who had successful balloon angioplasty. It was concluded that echo-Doppler studies are useful in demonstrating effectiveness of relief of aortic obstruction by balloon angioplasty, but are not predictive of exact residual gradients. This prompted us to conduct more extensive study to evaluate this issue [18], which will be discussed in the next section.

## 17. Value of Doppler in the Prediction of Pressure Gradients across Coarctation of the Aorta

**Figure 55:**
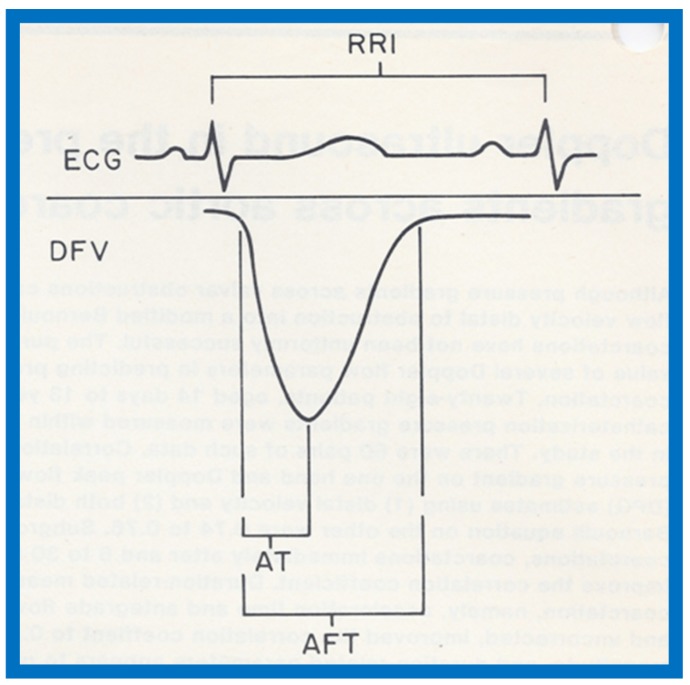
Line drawing of the Doppler flow velocity curve sampled from the descending aorta distal to coarctation site from a suprasternal notch view demonstrating measurement of duration-related parameters. AFT: antegrade flow time; AT: acceleration time; ECG: electrocardiogram; DFV: Doppler flow velocity curve; RRI: R-R interval of the ECG. Reproduced from Rao P.S., Carey P. [18].

Given the inability of Doppler to predict gradients across the coarctation site, as described in the preceding study [17] and reports with contradictory findings [51,52], with the former suggesting excellent correlation [51] and the latter indicating poor relationships [52], we decided to examine this issue with a study involving a sizable group of patients with aortic coarctation [18]. Sixty pairs of data sets with Doppler variables and catheterization-measured pressure gradients recorded within 24 h of each other from twenty-eight patients, aged 14 days to 13 years (4.5 ± 3.9 years), were included in the study [18]. Doppler pressure gradients were estimated using distal velocity alone (∆P = 4V^2^) and both distal and proximal velocities (∆P = 4[V_2_^2^ − V_1_^2^]) in the modified Bernoulli equation. Duration-related measures of Doppler flow signal distal to the coarctation site, namely, acceleration time (AT) and antegrade flow time (AFT) (Figure 55), were also measured. 

**Figure 56:**
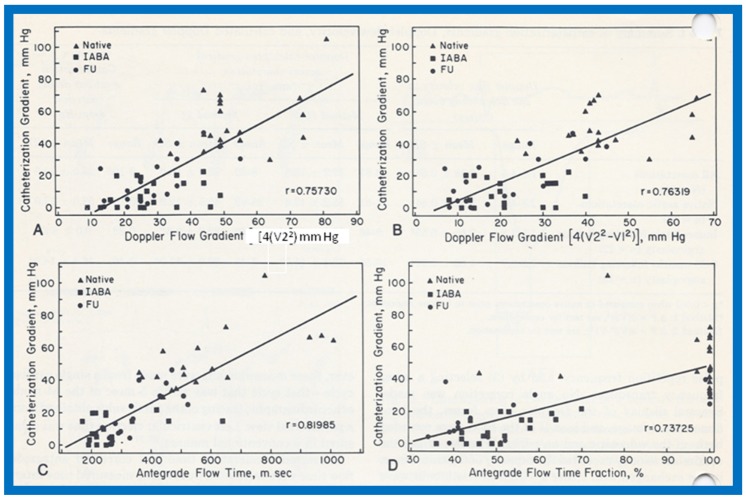
Linear regression analysis of catheterization-measured peak-to-peak and Doppler-derived (modified Bernoulli equation) peak instantaneous gradients across aortic coarctation are shown in (**A**,**B**). Note the similar correlation coefficients irrespective of the inclusion of proximal Doppler velocities. Similar regression analysis of catheterization-measured peak-to-peak gradients and antegrade flow time (milli seconds) (**C**) and antegrade flow time fraction (%) (**D**) show minimal increase in correlation coefficient (*r* = 0.82) when antegrade flow time is used. Filled triangles: Native coarctations; Filled squares: coarctations immediately after balloon angioplasty (IABA); Filled circles: coarctations at follow-up (FU). Reproduced from Rao P.S., Carey P. [18].

**Figure 57:**
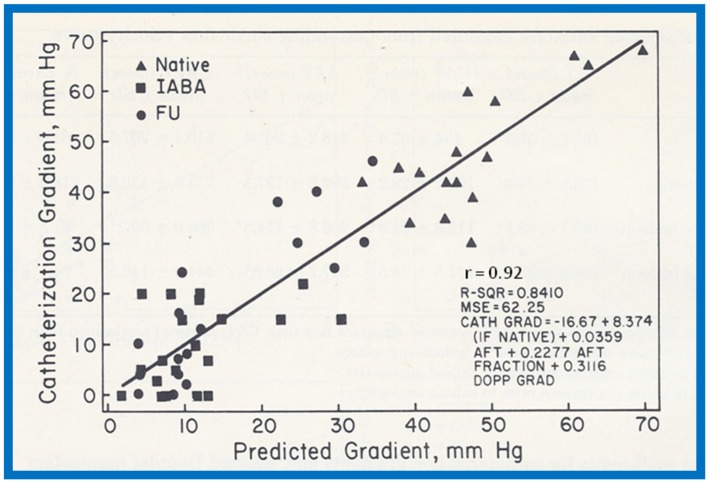
Linear regression analysis of catheterization-measured peak-to-peak gradients across aortic coarctation and predicted gradient calculated by the formula shown in the text indicated a better correlation (*r* = 0.92). Filled triangles: Native coarctations; Filled squares: coarctations immediately after balloon angioplasty (IABA); Filled circles: coarctations at follow-up (FU). Reproduced from Rao P.S., Carey P. [18].

**Figure 58:**
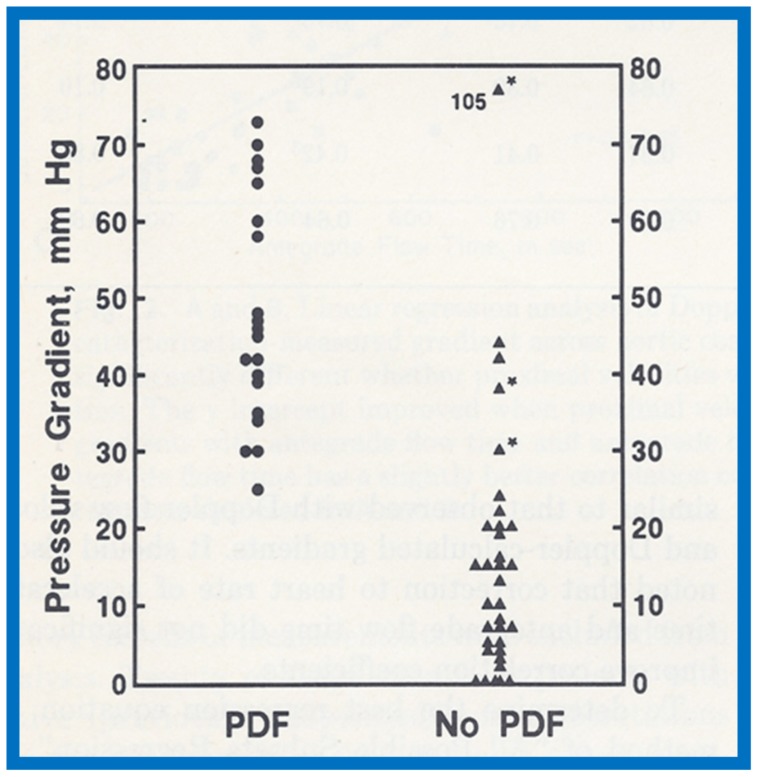
Catheterization-measured peak-to-peak gradients across aortic coarctation is expressed as a function of descending aortic pandiastolic flow (PDF) distal to the coarctation site. All patients with PDF (filled circles) had gradients of 25 mmHg or more. All but five patients without PDF (filled triangles) had gradients less the 25 mmHg. Three (marked with asterisk) of these five patients had long segment coarctations. Reproduced from Rao P.S., Carey P. [18].

A comparison of the Doppler-derived (∆P = 4V^2^) peak instantaneous and catheterization-measured peak-to-peak gradients resulted in a correlation coefficient of 0.757. (Figure 56A). The inclusion of both proximal and distal velocities (∆P = 4[V_2_^2^ − V_1_^2^]) in the modified Bernoulli equation (Figure 56B) did not significantly increase the correlation (*r* = 0.763). A comparison of the catheterization data with antegrade flow time (*r* = 0.82) and antegrade flow time fraction (*r* = 0.737) showed marginal, if any, improvement in the correlation coefficients (Figure 56C,D). Separating into subgroups of native, immediate postballoon and follow-up postballoon did not improve the correlation coefficients (Tables II and III from [18]). However, a blend of magnitude and duration related Doppler curves (using the method of “All Possible Subsets Regression”; Catheterization gradient = 0.31DPV + 0.22 AFT fraction + 0.04 AFT − 16.67) [18] improved the correlation coefficient to 0.92 (Figure 57). It was also observed that peak gradients in excess of 25 mmHg were seen in the presence of pandiastolic flow in the descending aortic Doppler curve with 100% specificity and 80% sensitivity (Figure 58). 

It was further noted that the Doppler flow velocity magnitude and calculated gradients improved (*p* < 0.001) following balloon angioplasty of native aortic coarctation; this was similar to reduction in peak-to-peak pressure gradients by catheterization. A discussion of possible reasons for the lack of correlation between Doppler and catheter gradients included peak-to-peak vs. peak instantaneous gradients and possible collateral vessel flow in aortic coarctation. It was concluded that (i) the magnitude and duration related Doppler velocity recordings are helpful in estimating coarctation gradients, and (ii) Doppler is useful in demonstrating the relief of obstruction by balloon angioplasty [18]. 

## 18. Foramen Ovale and Transatrial Doppler Velocity Patterns in the Normal Fetus

**Figure 59:**
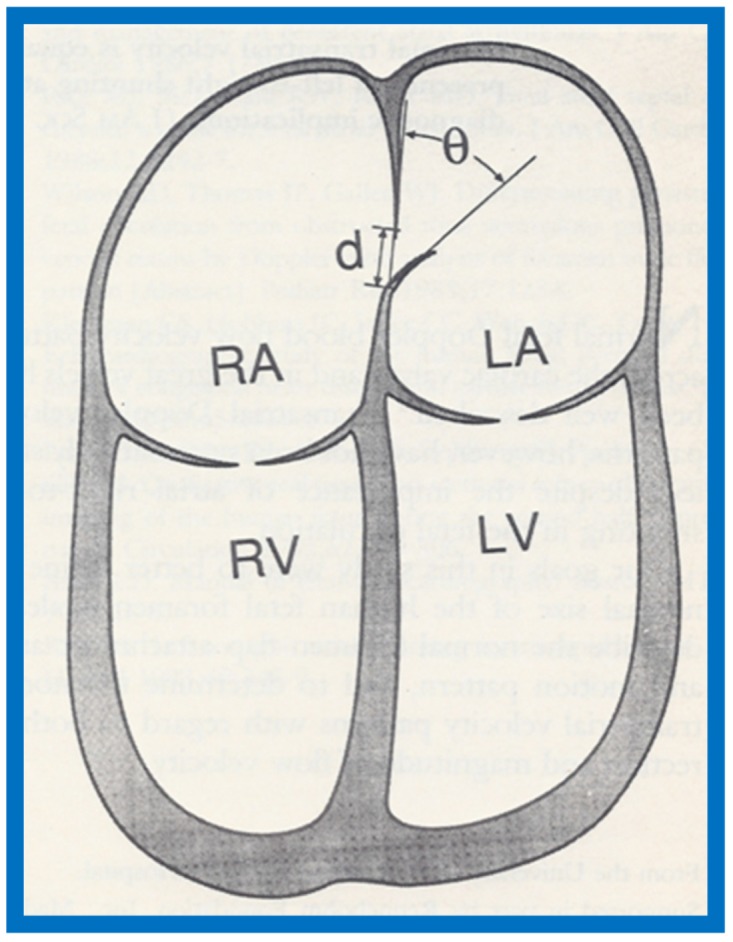
Drawing of the fetal heart demonstrating how the measurements are secured. Diameter (d) of the patent foramen ovale, as measured is shown. The angle (θ) is measured at the origin of the flap of foramen ovale with the atrial septum. The angle varied between 30 and 50 degrees; it was at least 30 degrees in all fetuses. LA: left atrium; LV: left ventricle; RA: right atrium; RV: right ventricle. Reproduced from Wilson AD, Rao P.S., Aeschlimann S. [19].

Despite the importance of foramen ovale (FO) in fetal circulation, the size of and shunt velocity patterns across the FO in the fetus had not been thoroughly investigated as of the mid-/late-1980s. Therefore, we endeavored to define the normal size of FO and the Doppler velocity patterns across the atrial septum in the normal fetus. Normal human fetal ultrasound studies in 48 consecutive fetuses were examined and the size of FO, foramen flap angle (Figure 59), its motion and flow characteristics across it were scrutinized [19]. 

**Figure 60:**
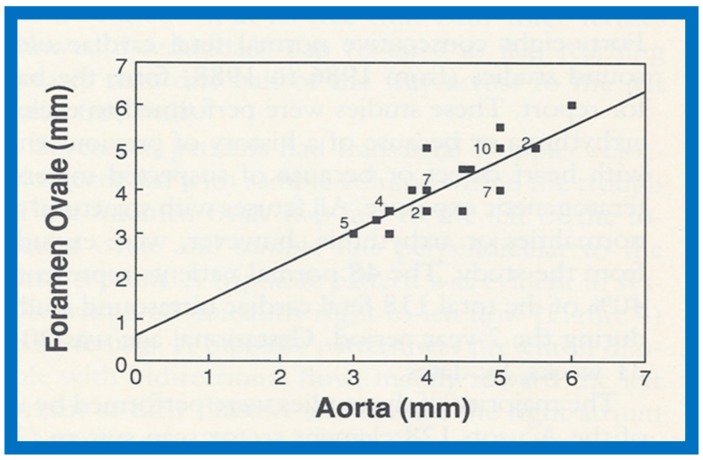
Plot of the diameter of the foramen ovale against diameter of the aorta. The numbers indicate the number of subjects with that particular measurement. Note excellent correlation with an *r* value of 0.84, y intercept of 0.605 and slope of 0.817. Reproduced from Wilson A.D., Rao P.S, Aeschlimann S. [19].

**Figure 61:**
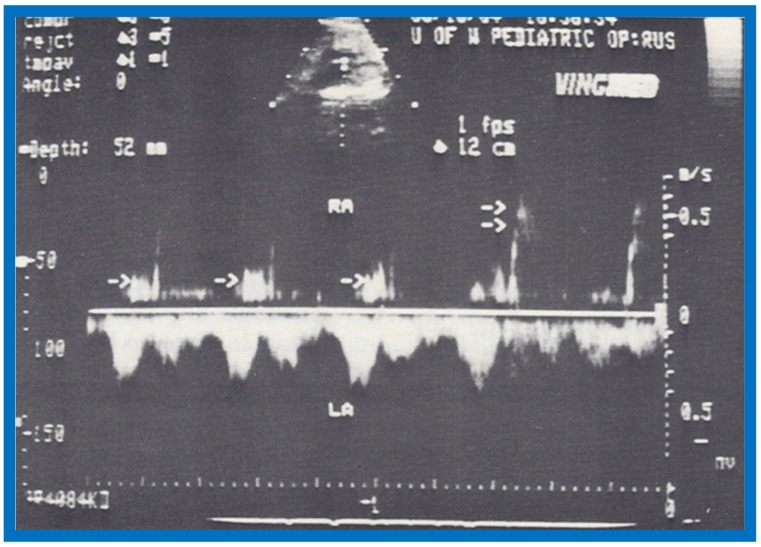
Pulse Doppler recording with the sample volume placed on the left atrial side of the foramen ovale (insert at the top) demonstrating transatrial flow. The majority of the flow is from the right atrium (RA) to the left atrium (LA) (bottom of the baseline). Left-to-right atrial shunt (above the baseline) is also seen, as marked by single arrows in the first three beats. Double arrows on the fourth beat shows Doppler recording of the ascending aortic flow, which serves to reference the timing of ventricle systole. Reproduced from Wilson AD, Rao P.S., Aeschlimann S. [19].

The maximal FO diameter was similar to the diameter of the Ao at all gestational ages (Figure 60); there was no more than 1.0 mm difference between the size of FO and Ao in any study. When the size of FO is expressed as a percentage (%) of aortic root, it was 96%, with a range of 77% to 125%. The angle of attachment of foramen flap at the junction of FO with the rim of the FO varied from 30° to 50° degrees; a minimum of 30° angle was seen at some point in the cardiac cycle. A redundant FO flap was seen in only three fetuses. The transatrial Doppler flow velocities with sample volume placed across FO on the left atrial side of the FO (Figure 61) were available for review in seventeen patients. A triphasic pattern was seen in systole with predominantly right-to-left flow (right atrium [RA] to LA) in all fetuses (Figure 61). Right-to-left flow velocity varied from 15 to 40 cm/s with a mean of 23 cm/s, and was always less than or equal to mitral valve A wave velocity. Left-to-right flow was also seen in late diastole in all studies (Figure 61; single arrows in the first three beats). The left-to-right flow was also documented by color flow imaging in some of these babies. The left-to-right flow velocity was lower and ranged between 5 to 20 cm/s with a mean of 13 cm/s. 

Our study showed that the size of normal foramen ovale is similar to aortic root diameter, and that the foramen flap angle reaches 30 degrees or greater. Right-to-left flow is as anticipated for fetal circulatory physiology; the left-to-right flow documented in this study was unexpected and may be related instantaneous pressure difference between atria, as previously shown in patients with tricuspid atresia [53]. These finding of left-to-right flow across the foramen ovale may have implications in conditions such as total anomalous pulmonary venous connection and severe left heart obstructive lesions [19].

The author’s contributions to the echocardiography literature from 1991 to the current date will be detailed in Part II of this review.

## Figures and Tables

**Table 1:** Left Ventricular Function Parameters Examined.

Parameter	How Derived
Systolic Time Intervals (Figure 11 and Figure 12)	
Preejection Period [PEP]	Onset of QRS to opening of the aortic valve
Left Ventricular Ejection Time [LVET]	Opening to closure of the aortic valve
Isovolumic Contraction Time [ICT]	PEP–QMc
PEP/LVET ratio	Ratio of PEP to LVET
Left Ventricular Volumes	
LV End-Diastolic Volume [LVEDV]	LVLDV = 1.05 × LVEDD3
LV End-Systolic Volume [LVESV]	LVLSV = 1.05 × LVESD3
Stroke Volume (SV)	SV = LVEDV − LVESV
Ejection Fraction (EF)	EF = SV/LVEDV
Cardiac Output (COP)	COP = SV × Heart Rate
Shortening Fraction (Figure 13)	SF = ([LVEDD − LVESD]/LVEDD) × 100
Velocity of Circumferential Fiber Shortening (Vcf)	Vcf = (LVEDD − LVESD)/(LVEDD × LVET)
Percent Thickening of LV Posterior Wall	([LV posterior wall thickness in end systole − LV posterior wall thickness in end diastole]/LV posterior wall thickness in end diastole) × 100)

LV: left ventricle; LVEDD: LV end-diastolic dimension; LVESD: LV end-systolic dimension; QMc: Q wave of the ECG to closure of the mitral valve (Figure 13B).

**Table 2:** Left Ventricular Muscle Mass by Echocardiography.

Patient Group	LVMM(C) g/M^2^	*p*-Value	LVMM(T) g/M^2^	*p*-Value	LVMM(R) g/M^2^	*p*-Value
LV Muscle	Mass in	Diastole				
White	89 ± 18		121 ± 20		52 ± 25	
Black	96 ± 17	<0.05	130 ± 20	<0.01	54 ± 24	>0.1
Sickle cell disease	170 ± 44	<0.001	202 ± 50	<0.001	131 ± 46	<0.001
LV Muscle	Mass in	Systole				
White	88 ± 15		75 ± 11		30 ± 17	
Black	90 ± 19	>0.1	77 ± 13	>0.1	38 ± 21	<0.01
Sickle cell disease	163 ± 47	<0.001	128 ± 35	<0.001	77 ± 35	<0.001

C: cubed-function method; g/M^2^: grams per square meter of body surface area; LV: left ventricle; LVMM: LV muscle mass; R: Ratshin method; T: Teichholz method. Reproduced from Rao P.S., et al. [9].
